# A mistletoe tale: postglacial invasion of *Psittacanthus schiedeanus* (Loranthaceae) to Mesoamerican cloud forests revealed by molecular data and species distribution modeling

**DOI:** 10.1186/s12862-016-0648-6

**Published:** 2016-04-12

**Authors:** Juan Francisco Ornelas, Etelvina Gándara, Antonio Acini Vásquez-Aguilar, Santiago Ramírez-Barahona, Andrés Ernesto Ortiz-Rodriguez, Clementina González, María Teresa Mejía Saules, Eduardo Ruiz-Sanchez

**Affiliations:** Departamento de Biología Evolutiva, Instituto de Ecología, A.C., Carretera antigua a Coatepec No. 351, El Haya, Xalapa, Veracruz 91070 Mexico; Department of Plant and Microbial Biology & The University and Jepson Herbaria, University of California, Berkeley, 431 Koshland Hall, Berkeley, CA 94270 USA; Cátedras CONACYT-Instituto de Investigaciones sobre los Recursos Naturales, Universidad Michoacana de San Nicolás de Hidalgo, Av. San Juanito Itzícuaro s/n, Col. Nueva Esperanza, Morelia, Michoacán CP 58330 Mexico; Centro Regional del Bajío, Instituto de Ecología, A.C., Avenida Lázaro Cárdenas 253, Pátzcuaro, Michoacán 61600 Mexico

**Keywords:** Loranthaceae, Mesoamerica, Mistletoes, Phylogeography, Pleistocene, *Psittacanthus*

## Abstract

**Background:**

Ecological adaptation to host taxa is thought to result in mistletoe speciation via race formation. However, historical and ecological factors could also contribute to explain genetic structuring particularly when mistletoe host races are distributed allopatrically. Using sequence data from nuclear (ITS) and chloroplast (*trnL-F*) DNA, we investigate the genetic differentiation of 31 *Psittacanthus schiedeanus* (Loranthaceae) populations across the Mesoamerican species range. We conducted phylogenetic, population and spatial genetic analyses on 274 individuals of *P. schiedeanus* to gain insight of the evolutionary history of these populations. Species distribution modeling, isolation with migration and Bayesian inference methods were used to infer the evolutionary transition of mistletoe invasion, in which evolutionary scenarios were compared through posterior probabilities.

**Results:**

Our analyses revealed shallow levels of population structure with three genetic groups present across the sample area. Nine haplotypes were identified after sequencing the *trnL-F* intergenic spacer. These haplotypes showed phylogeographic structure, with three groups with restricted gene flow corresponding to the distribution of individuals/populations separated by habitat (cloud forest localities from San Luis Potosí to northwestern Oaxaca and Chiapas, localities with xeric vegetation in central Oaxaca, and localities with tropical deciduous forests in Chiapas), with post-glacial population expansions and potentially corresponding to post-glacial invasion types. Similarly, 44 ITS ribotypes suggest phylogeographic structure, despite the fact that most frequent ribotypes are widespread indicating effective nuclear gene flow via pollen. Gene flow estimates, a significant genetic signal of demographic expansion, and range shifts under past climatic conditions predicted by species distribution modeling suggest post-glacial invasion of *P. schiedeanus* mistletoes to cloud forests. However, Approximate Bayesian Computation (ABC) analyses strongly supported a scenario of simultaneous divergence among the three groups isolated recently.

**Conclusions:**

Our results provide support for the predominant role of isolation and environmental factors in driving genetic differentiation of Mesoamerican parrot-flower mistletoes. The ABC results are consistent with a scenario of post-glacial mistletoe invasion, independent of host identity, and that habitat types recently isolated *P. schiedeanus* populations, accumulating slight phenotypic differences among genetic groups due to recent migration across habitats. Under this scenario, climatic fluctuations throughout the Pleistocene would have altered the distribution of suitable habitat for mistletoes throughout Mesoamerica leading to variation in population continuity and isolation. Our findings add to an understanding of the role of recent isolation and colonization in shaping cloud forest communities in the region.

**Electronic supplementary material:**

The online version of this article (doi:10.1186/s12862-016-0648-6) contains supplementary material, which is available to authorized users.

## Background

Most aerial Loranthaceae mistletoes use bird vectors for seed dispersal and, eventually, branch attachment and penetration of woody host plants [1]. Given their close affinities with their dispersal vectors and the connections with and dependence on their hosts [2, 3], the mistletoe geographical ranges are directly related to the morphological and behavioral adaptations of mistletoe fruit specialists and the availability of suitable host trees. However, most avian vectors of mistletoe parasites do not seem to be sufficiently specialized to isolate mistletoe populations on different hosts ([4] and references therein) and, therefore, the genetic structuring of mistletoe populations is likely more influenced by the geographic structuring of host populations [5–7].

Research on mistletoe evolution has highlighted the importance of host specialization during speciation (reviewed in [5]), which can occur in three ways: (1) mistletoe co-speciation with their hosts (*dispersal hypothesis*), (2) mistletoe speciation by changing host specificity (*host-switching hypothesis*), and (3) mistletoe speciation without changes in the host (*ecological hypothesis*). Each of these modes of mistletoe speciation could occur following the allopatric, peripheral isolates or sympatric models of speciation [8]. *Psittacanthus* Mart. (ca. 120 species; [3, 9]) mistletoes show a variety of patterns of host specificity from specialist to generalist [10]. For example, three species with allopatric distribution in Mesoamerica, *P. angustifolius* (Chiapas to Nicaragua)*, P. macrantherus* (Trans-Mexican Volcanic Belt and Sierra Madre Occidental) and *P. pinicola* (Atlantic coast from Belize to Nicaragua), parasitize exclusively species of *Pinus* and *Abies* [11, 12], while *P. sonorae* (Sonora and Baja California Sur, Mexico), *P. nudus* (Yeguare Valley, Honduras) and *P. palmeri* (along the Trans-Mexican Volcanic Belt) parasitize almost exclusively species of *Bursera*, and *P. auriculatus* (Tehuacan-Cuicatlán Valley, Mexico) and *P. breedlovei* (Chiapas Central Depression, Mexico) parasitize *Acacia* species and other legumes [3, 13]. In contrast, other *Psittacanthus* species widely distributed in Mesoamerica often in sympatry parasitize distantly related host species (e.g., *P. schiedeanus*, *P. mayanus*, *P. calyculatus*, *P. ramiflorus*; [3, 14, 15]). That is, there is a tendency among *Psittacanthus* mistletoes that infect more than one host species to infect distantly related hosts and their ranges overlap, whereas *Psittacanthus* species that infect closely related host species have allopatric distributions.

The tendency for closely related mistletoes to infect closely related host species supports the model of mistletoe co-speciation with their hosts (*dispersal hypothesis*). In contrast, host specialization might play an important role in speciation to occur among species of mistletoes using several host species, and mistletoe speciation in sympatry would occur by changing host specificity (*host-switching hypothesis*) leading to specialization on different hosts and the formation of mistletoe races. If a mistletoe species utilizes two or more host species, the genetic pool of the mistletoe species may become differentiated into “host races,” which may eventually diverge into new species (i.e., follow a model of speciation by host race formation).

The existence of host-specific relationships through cross-dispersal experiments has been suggested as evidence for the presence of host-specific races in a number of mistletoe species (e.g., [15–21]), or by using genetic markers, including isozymes [22–26], AFLPs [27] and chloroplast DNA sequences [6, 7, 28, 29]. Cross-dispersal experiments have shown that seedling development is greatest when mistletoe seeds are placed on their source host species [15–17, 20, 21, 30, 31]. Using genetic data, however, the distinct host races identified were geographically isolated by some distance (except [25, 26]) or the phylogeographic groups were best explained by isolation linked to Pliocene geological events and Pleistocene climate changes [6, 7], which makes it difficult to distinguish between the potential effects of isolation and those linked to changes in host-tree availability on population divergence.

A phylogeographic approach is appropriate for evolutionary studies of mistletoes, because the assessment of geographic structure and genetic variation within and among populations might reflect underlying spatial and/or temporal discontinuities in available host tree species. Despite the appeal of the speciation model of host race formation by host switching, species formation in mistletoes that are transmitted between hosts by avian vectors is not understood. Speciation via race formation is thought to be the result of ecological adaptation to host taxa, and presumably the evolutionary pathway that eventually lead to mistletoe diversification [5]. However, other factors could also contribute to explain genetic structuring of mistletoe populations particularly when mistletoe “host races” are distributed allopatrically. It may occur when gene flow between conspecific populations is diminished because of the synergistic effects of both paleogeographical events and Pleistocene glacial-interglacial cycles that, coupled with the complex interactions between mistletoes and their tree hosts, pollinators and seed dispersers, could have shaped at different time scales the distribution and genetic structure of species in a region (e.g., [32]).

Here the evolutionary history of *Psittacanthus schiedeanus* (Schltdl. & Cham.) G. Don (Loranthaceae) is examined through species distribution modeling and phylogeographic and population genetic analyses of nuclear and chloroplast DNA. We used a broad geographical sampling of populations of *P. schiedeanus* DNA sequences from the internal transcribed spacer (ITS) region of the nuclear ribosomal DNA (nrDNA) in combination with sequences of the rapidly evolving *trnL-F* intergenic spacer region from the chloroplast genome (cpDNA). This multilocus approach allowed us to explicitly test the set of historical and climatic scenarios in the evolution of *P. schiedeanus* and identify patterns of population structure that may reflect the evolutionary history of the species. As the chloroplast genome is maternally inherited in most angiosperms, cpDNA sequences may provide a picture of evolutionary haplotype relationships of seed-mediated gene flow compared to the ITS sequences reflecting a combination of both pollen and seed movement [33, 34]. Specifically, we address the following questions: (1) Does the phylogeography of *P. schiedeanus* based on nuclear and chloroplast markers support a vicariance model in which major geographic disjunctions (in particular the Isthmus of Tehuantepec break) or historical fragmentation of cloud forest distribution correspond with genetic diversification? (2) Are the phylogeographic patterns concordant between the two types of markers? (3) Does niche modeling also support the vicariance model, and does the proposed patterns of suitable habitat distribution though Pleistocene glacial-interglacial cycles match those suggested by phylogeography? And (4), Does the phylogeography of *P. schiedeanus* support the model of ecological speciation due to host-specificity, and does this reflect the observed host population expansion/contraction events historically related to the availability of suitable host trees?

## Methods

### Study system

*Psittacanthus schiedeanus* mistletoes have yellow-to-orange, self-compatible hermaphroditic flowers pollinated mainly by hummingbirds [35], and ripe purplish-black, lipid-rich, fleshy (one-seed) fruits dispersed by a variety of birds [14, 20, 36, 37]. These mistletoes are characteristic of the canopy in the cloud forests edges in northern Mesoamerica, here delineated from northeastern Mexico to the Guatemalan highlands [38]. The northern boundary of the region comprises cloud forests along the Sierra Madre Oriental, from southern Tamaulipas to northern Oaxaca. The southern boundary includes cloud forests in the Sierra Madre de Chiapas, central highlands of Chiapas, and in the Sierra de Las Minas and Sierra de Cuchumatanes in Guatemala (Additional file 1). Between these boundaries, cloud forests encompass an extremely heterogeneous mixture of North American temperate tree species that are present in the region since the Tertiary and tropical species with South American origins during the early Miocene [32, 39–42].

*Psittacanthus schiedeanus* often parasitizes more than 20 host tree species, introduced to or native to cloud forests [43–45]. In central Veracruz, the most severe infections occur on *Liquidambar styraciflua* [14, 20, 45], a temperate tree species present in Mesoamerica as early as the Late Miocene [42, 46]. A phylogeographic study in *L. styraciflua* revealed breaks between populations in the deciduous forests of the southeastern USA and the Mesoamerican cloud forests, populations distributed north and south of the Trans-Mexican Volcanic Belt (TMVB), and private haplotypes for populations in the cloud forest of the Sierra de Los Tuxtlas [46]. The genetic divergence between *L. styraciflua* populations distributed north and south of the TMVB occurred 4.27–1.42 million years ago (MYA) during the formation of the Pliocene to Quaternary volcanic arc, one of the most recent episodes of the geologic evolution of the TMVB (at ca. 3.6 MYA), and subsequent climatic changes in the region. Ruiz-Sanchez and Ornelas [46] also found signals of demographic expansion during the Last Glacial Maximum (LGM, ca. 20,000 years ago) in the Mesoamerican populations of *L. styraciflua* that presumably connected and expanded to lower coastal areas. Given that the geographic structuring of host populations must influence the genetic structuring of mistletoe populations, the current distribution range and phylogeographic structure of *P. schiedeanus* populations should reflect the observed host population expansion/contraction events historically related to the availability of suitable host trees. However, this hemiparasitic mistletoe may not track their *L. styraciflua* hosts during range expansions and contractions linked to Pleistocene climate changes because it lives on several host tree species (non-obligate host-parasite relationship) and, as a consequence, its distribution range was not influenced by geographical host range changes during glacial/interglacial periods.

### Samples and DNA sequencing

Leaf tissue samples were collected from 274 individuals on a variety of host tree species in 31 populations throughout the species range in Mexico (Additional files 1 and 2). Target sampling localities were chosen based on localities of occurrence data acquired from Kuijt [3]. *Psittacanthus schiedeanus* can be relatively easy to identify in the summer time because of its conspicuous and long flowers. However, it is an extremely variable species in flower and leaf morphology sometimes difficult to separate from sympatric *P. calyculatus* [3], even though its typical form west of the Isthmus of Tehuantepec has much longer and more slender flowers [35, 47] and *P. angustifolius* with narrow leaves similar to occasional individuals of *P. schiedeanus* but prefers *Pinus chiapensis* as host at higher elevations in Chiapas and *Pinus oocarpa* in Honduras [3, 48], and from *P. mayanus* (Yucatan Peninsula), *P. breedlovei* (Chiapas) and *P. minor* (northern Nicaragua) allopatrically distributed in tropical deciduous forests mainly on *Acacia* host trees at lower elevations [3]. Therefore, our geographic sampling scheme includes populations considered phenotypically *P. schiedeanus* along the cloud forest distribution and samples phenotypically similar to *P. schiedeanus* of adjacent populations regardless of taxonomy, focusing on those distributed in central Oaxaca (*P. calyculatus*) and Chiapas (*P. breedlovei*). Most populations collected have an accompanying voucher that is deposited at the XAL herbarium of the Instituto de Ecología, AC (INECOL) (Additional file 2). Only one *P. schiedeanus* plant was sampled per individual host tree.

Leaf tissue samples were preserved in silica gel desiccant until DNA extractions were performed. We additionally obtained leaf samples for DNA sequencing from other *Psittacanthus* species (Additional file 3) and downloaded DNA sequences from the GenBank of representatives of Loranthaceae tribes to be used as outgroups: *Nuytsia floribunda* (DQ333867, DQ788716), *Atkinsonia ligustrina* (DQ333865, DQ788714), *Gaiadendron punctatum* (DQ333866, DQ340617), *Peraxilla tetrapetalla* (DQ333846, DQ340597), *Alepis flavida* (DQ333847, DQ340598), *Desmaria mutabilis* (DQ333852, DQ340603), *Phthirusa pyrifolia* (DQ333857, EU544504), *Oryctanthus occidentalis* (DQ333862, DQ340613), *Tupeia antarctica* (DQ333850, DQ340601), *Phragmanthera regularis* (DQ333830, DQ340579), *Tristerix corymbosus* (DQ333854, DQ340605)*, Tristerix aphyllus* (DQ442966, DQ442919), *Tripodanthus acutifolius* (DQ333864, DQ340615), *Notanthera heterophylla* (DQ333855, DQ340606), *Ligaria cuneifolia* (DQ333853, DQ340604), *Cladocolea cupulata* (DQ333861, DQ340612), *Cladocolea mcvaughii* (DQ333860, DQ340611) and *Struthanthus orbicularis* (DQ333856, DQ340607) from Wilson and Calvin [49], Amico et al. [50] and Vidal-Russell and Nickrent [51].

Total genomic DNA was extracted from silica-dried material using a modified 2 × cetyl trimethyl ammonium bromide (CTAB) protocol [52] or the DNeasy Plant Mini kit (Qiagen, Valencia, CA, USA) using the manufacturer protocol. Amplification of the nrDNA ITS region was conducted with the primers ITS5HP [53] and ITS4 [54], whereas for *trnL-F* intergenic spacer region we used the universal primers e and f [55]. For targeting successful of ITS region sequencing, we used the primers ITS-F2-Psitta (5′-TCGCAGTATGCTCCGTATTG-3′) and ITS-R2-Psitta (5′-TCGTAACAAGGTTTCCGTAGG-3′) designed for this study. The 25–μL ITS PCR mix contained 5 μL of 5 × buffer (Promega, Madison, WI, USA), 2.0 μL MgCl_2_ (25 mM), 2 μL dNTPs mix (8 mM), 0.82 μL of each primer (10 μM), 0.27 μL Taq polymerase (5U/μL) (Promega), 2 μL of DMSO (Sigma), 1.5–3 μL of template DNA, and finally dH_2_O added to bring to volume. The 25–μL *trnL-F* PCR mix contained 3.5 μL of 5 × buffer (Promega, Madison, WI, USA), 3.5 μL MgCl_2_ (25 mM), 2 μL dNTPs mix (8 mM), 0.45 μL of each primer (10 μM), 0.20 μL Taq polymerase (5U/μL) (Promega), 1.5–3 μL of template DNA, and finally dH_2_O added to bring to volume. PCRs of ITS consisted of an initial denaturation at 94 °C for 4 min, followed by 5 cycles of 94 °C for 1 min, 50 °C for 30 s, 72 °C for 1 min, followed by 30 cycles of 94 °C for 30 s, 45–52 °C for 30 s, 72 °C for 1 min, and a final step of 72 °C for 7 min. Chloroplast (*trnL-F*) amplifications used the following profile: initial denaturation at 95 °C for 5 min, followed by 40 cycles of 95 °C for 10 s, 55 °C for 1 min, 72 °C for 20 s, and a final step of 72 °C for 7 min. PCR products were purified with the QIAquick kit (Qiagen) and sequenced in both directions to check the validity of the sequence data using the BigDye Terminator Cycle Sequencing kit (Applied Biosystems, Foster City, California, USA). The products were analyzed on a 310 automated DNA sequencer (Applied Biosystems) at the INECOL’s sequencing facility, or at University of Washington High Throughput Genomics Unit, Seattle, Washington. Finally, sequences were assembled using Sequencher v4.9 (Gene Codes, Ann Arbor, MI, USA) and then were manually aligned with SE-AL v2.0a111 (http://tree.bio.ed.ac.uk/software/seal). All unique sequences of *P. schiedeanus* and those new of other *Psittacanthus* species have been submitted to GenBank (Accession nos. ITS: KU922961–KU923004, KU923036–KU923037; *trnL-F*: KU923270–KU923278, KU923310–KU923311; Additional file 3).

### Paleodistribution modeling and environmental variation

We used species distribution modeling (SDM; [56]) to predict where populations of *P. schiedeanus* resided at the LGM (ca. 20,000 years ago) and Last Inter Glacial (LIG; ca. 130,000 years ago), and whether range expansion and population connectivity are observed according to the predicted changes in the distribution of cloud forests [57, 58]. Coordinates of occurrence data were assembled for *P. schiedeanus* obtained from the Global Biodiversity Information Facility (GBIF; http://www.gbif.org/species/4003073), supplemented with records from field collection (see dataset on Dryad: doi:10.5061/dryad.t49h6). After careful verification of every data location and removing duplicate occurrence records, we restricted the data sets to unique records for the analyses, leaving 58 unique presence records. Distributional records were input into and analyzed to infer a SDM with the maximum entropy algorithm in MAXENT v3.2.2 [59] and using the ArcView v3.2 (ESRI, Redlands, CA, USA) to extract the GIS data. Present-day temperature and precipitation data (BIO 1–19 variables) were drawn as climate layers from the WorldClim database at a spatial resolution of ca. at 1 km^2^ in each raster ([60]; http://www.worldclim.org). We first constructed a model to the present using all climatic variables with 80 % of the locality records as training data and the other 20 % as testing data in addition to response curves, jackknife tests, logistic output format and random seed parameters. Variable importance was determined comparing percentage contribution values and jackknife plots. Then, correlation coefficients between present variables were calculated using JMP v5.1 (SAS Institute Inc. Cary, NC, USA) with the objective to identify and remove the variables that were highly correlated (correlation values ≥0.8) and less explanatory, having as a rule of decision, the relative contributions of each of them in the MaxEnt model. After removing the highly correlated variables, five were used in the final analyses: Temperature Seasonality (BIO4), Min Temperature of Coldest Month (BIO6), Temperature Annual Range (BIO7), Precipitation of Wettest Month (BIO13) and Precipitation Seasonality (BIO15). Final models were constructed with 10 cross-validation replicates without extrapolation and considering the average output grids as the final predictive models. The area under the receiver operating characteristic curve (AUC) was used to evaluate the prediction performance of the models, where 1 is the maximum prediction and 0.5 suggest a random prediction. Resulting species distributions under current climate conditions were projected onto past climate scenarios, at the LGM (at 2.5 arc-minutes) and LIG (at 30 arc-seconds). Past climate layers were also drawn from the WorldClim webpage for two LGM scenarios developed by the Palaeoclimate Modelling Intercomparison Project Phase II [61]: the Community Climate System Model (CCSM; [62]) and the Model for Interdisciplinary Research on Climate (MIROC; [63]), and the LIG [64]. The CCSM and MIROC climate models simulate different climate conditions, with cooler sea-surface temperature conditions assumed in CCSM than in MIROC, resulting in higher annual precipitation in CCSM than in MIROC [58, 65, 66].

Measurements of temperature and precipitation (BIO 1–19 variables) were extracted for each of the sampling locations from WorldClim [60] as explained above. We performed principal components analysis (PCA) using SPSS v17 (SPSS, Armonk, NY, USA) to reduce intercorrelated BIO variables to a smaller system of uncorrelated, independent variables, and used the resulting PC loadings to examine habitat and ecological variation potentially related to population divergence.

### Phylogenetic analysis, divergence time estimation and haplotype relationships

Phylogenetic relationships among sequences based on Bayesian Inference (BI) were reconstructed using MRBAYES v3.12 [67, 68]. BI analyses were run using the CIPRES Science Gateway [69] for the ITS, *trnL-F* and the concatenated data sets (see ITS and *trnL-F* datasets on Dryad: doi:10.5061/dryad.t49h6). jMODELTEST v0.1.1 [70] was run to choose the model of molecular evolution that best fitted our sequence data under the Bayesian information criterion (BIC), GTR+G, for the ITS, *trnL-F* and for the concatenated data set. Two parallel Markov chain Monte Carlo (MCMC) analyses were executed simultaneously, and each was run for 10 million generations, sampling every 1000 generations. Bayesian posterior probability values were calculated from the sampled trees remaining after 2500 samples were discarded as burn-in [67] to only include trees after stationarity was reached. The remaining trees were used to generate a 50 % majority-rule consensus tree, showing nodes with a posterior probability (PP) of 0.5 or more. We consider nodes significantly supported if posterior probabilities were ≥0.95 [67]. While *Struthanthus, Cladocolea* or *Aetanthus* may be the closest relatives of *Psittacanthus* [49, 51], relationships among phenotypically similar *Psittacanthus* species remain uncertain [3]. Thus, we included several congeners to root *P. schiedeanus* sequences. The likelihood scores under the ITS, *trnL-F* and the concatenated data sets were compared with ln Bayes factors (BF) tests to determine which tree topology significantly improved explanation of the data.

To relate genetic differentiation found among *P. schiedeanus* ribotypes and haplotypes to pre-Pleistocene and Pleistocene events, we estimated divergence time under a Bayesian approach as implemented in BEAST v1.6.1 [71] using the combined ITS and plastid *trnL-F* data set. The ingroup comprised all nrDNA or plastid cpDNA sequences of *P. schiedeanus* and sequences downloaded from GenBank of species representing Loranthaceae tribes from Amico et al. [50] and Vidal-Russell and Nickrent [51, 72] used as multiple outgroups. Divergence time estimation was performed in BEAST using the uncorrelated lognormal relaxed molecular clock and the closest nucleotide substitution model under the Bayesian information criterion (BIC), GTR+G for the concatenated data set, suggested by jMODELTEST. The tree prior model was set using a coalescent approach assuming constant population size. In this analysis, we constrained Nuytsiae as the sister group of members of other subtribes and *Psittacanthus* to be monophyletic based on Vidal-Russell and Nickrent [72] and the BI tree topology.

To calibrate the root node, the divergence time between *Nuytsia floribunda* Australian root parasite and aerial loranth parasites clade [72] was used as secondary calibration, approximating a median age of 28 MYA (normal distribution, mean 28, SD 3.7, range 34–21 MYA). The geometric mean of 5.798 × 10^−9^ substitutions per neutral site per year (s/s/y) was used to calibrate the tree based on the mean mutation rates of 4.13 × 10^−9^ s/s/y for ITS of herbaceous annual/perennial plants [73] and 8.24 × 10^−9^ s/s/y for the *trnL-F* estimated for annual or perennial herbs [74]. For divergence time estimation, BEAST was run two times for 10 million generations, sampling every 1000 steps. We combined log and trees files from each independent run using LOGCOMBINER v1.8.0 [71], then viewed the combined log file in TRACER v1.6 (http://tree.bio.ed.ac.uk/software/tracer/) to ensure that effective sample size (ESS) for all priors and the posterior distribution were >200, making sure that parameter values were fluctuating at stable levels. Based on these results, the first 10 % trees were discarded as burn-in, and the remaining trees were annotated and summarized as a maximum clade credibility tree with mean divergence times and 95 % highest posterior density (HPD) intervals of age estimates using TREEANNOTATOR v1.8.0 [71] and visualized in FIGTREE v1.3.1 (http://tree.bio.ed.ac.uk/software/figtree/).

To infer genealogical relationships among ribotypes (ITS) and haplotypes (*trnL-F*), statistical parsimony networks for both data sets were constructed as implemented in TCS v1.2.1 [75], with gaps treated as missing data and a connection limit set to 95 %. Loops were resolved following the criteria given by Pfenninger and Posada [76].

### Population genetic diversity indices, geographic structure and relationships among populations

Population diversity for unordered (*h*_S_, *h*_T_) and ordered haplotypes (*v*_S_, *v*_T_) and differentiation (*G*_ST_, *N*_ST_) parameters were estimated using PERMUT v1.0 [77]. Significant differences between *N*_ST_ and *G*_ST_ parameters were tested with 10,000 permutations. If *N*_ST_ is significantly larger than *G*_ST_, this could indicate that the haplotypes found in a given population are phylogenetically closely related [77]. We also calculated haplotype diversity (*h*), nucleotide diversity (π) and pairwise comparisons of *F*_ST_ values between groups (Additional file 2) in ARLEQUIN v3.01 [78] with 16000 permutations. Populations with three samples or fewer were excluded from this analysis.

To determine whether or not populations are structured by geography or habitat distribution, three analyses of molecular variance (AMOVAs; [79]) were performed based on pairwise differences using ARLEQUIN with populations treated as (a) a single group to determine the amount of variation partitioned among and within populations, and (b) grouped into east and west of the Isthmus of Tehuantepec, (c) three groups according to habitat distribution or (d) grouped into six groups according to geography and mountain range (Additional files 1 and 2). AMOVAs were performed using the Tamura-Nei model and 16,000 permutations to determine the significance of each AMOVA.

To estimate the relationships among groups of populations (Additional files 1 and 2), we used ITS and *trnL-F* sequences for *P. schiedeanus* samples and *BEAST [80] with the multispecies coalescent model implemented in BEAST. *BEAST models the lineage sorting process between units for groups of individuals not connected by gene flow above, at, or below the species level [80]. *Psittacanthus mayanus* samples were used as outgroups. Nucleotide substitution models, JC for ITS and F81 for *trnL-F* selected with jMODELTEST were incorporated as the Hasegawa-Kishino-Yano (HKY) model for both markers. Species were assigned based on the identification of each sample using haplotype or ribotype distribution and locality. The simulation was first run with all samples as two lineages separated by the Isthmus of Tehuantepec (WEST and EAST), then with samples as six lineages corresponding to geography and mountain ranges (nSMO, cSMO, sSMO, CHIS, OAX, BREE) or three separate lineages according to habitat (SCHI = cloud forests from San Luis Potosí to Oaxaca and Chiapas, CALY = xeric vegetation in central Oaxaca, BREE = tropical deciduous forests in Chiapas). We ran each BEAST analysis two times for 30 million generations, sampling every 1000 steps, using a Yule speciation tree prior, relaxed clock model with an uncorrelated lognormal distribution, and the mean mutation rates of 4.13 × 10^−9^ s/s/y for ITS [73] and 8.24 × 10^−9^ s/s/y for the *trnL-F* [74]. After the analysis in BEAST, log and tree files were combined using LOGCOMBINER and summarized as a maximum clade credibility tree using TREEANNOTATOR with a burn-in of 25 %. We used TRACER to visualize the results of the runs and to check the ESS (cut off values >50) of each parameter.

### Demographic history

The demographic history of each *P. schiedeanus* resulting group was determined by means of neutrality tests and mismatch distributions carried out in ARLEQUIN. To test whether populations evolve under neutrality, Fu’s *Fs* test [81] and Tajima’s *D* [82] were calculated, and mismatch distributions [83] were calculated using the sudden expansion model of Schneider and Excoffier [84] with 1000 bootstrap replicates. The validity of the sudden expansion assumption was determined using the sum of squares differences (SSD) and Harpending’s raggedness index (Hri; [85]), both of which are higher in stable, non-expanding populations [86].

Bayesian skyline plots [87] were also used to infer changes in effective population size (*N*_e_) of each *P. schiedeanus* group (see Results) through time in BEAST. We chose a HKY substitution model with empirical base frequencies, a strict clock model, and a piecewise-linear coalescent Bayesian skyline tree prior with five starting groups. Two independent runs of 10 million generations each were run, with trees and parameters sampled every 1000-iterations, with a burn-in of 10 %. Results of each run were visualized using TRACER to ensure that stationarity and convergence had been reached, and that the ESS were higher than 50.

We used the software IMa [88] on *P. schiedeanus* genetic groups (see Results; see dataset on Dryad: doi:10.5061/dryad.t49h6) to estimate the effective population size of the ancestral (*q*_a_) and the two descendant populations (*q*_1_ and *q*_2_), effective number of migrants per generation in both directions (*m*_*1-to-2*_ and *m*_*2-to-1*_), and time since divergence (*t*) at which the ancestral population gave rise to the descendant populations. The isolation-with-migration (IM) model [89] is appropriate to estimate parameters for two descendant populations that have diverged recently from the ancestral population and that may be sharing haplotypes as a result of gene interchange. We began with multiple runs of 10,000 steps (following 100,000 iterations as burn-in) to assess mixing and to fine-tune the parameter space. We then conducted the simulation for a burn-in of 1 million generations and 30 million steps, under the HKY model of sequence evolution. Two independent runs were performed with different seed numbers to guarantee convergence of samples [89]. We considered that the analyses had converged upon a stationary distribution if the independent runs had similar posterior distributions and the ESS for each parameter was at least 50. We report the mean parameter estimates of two runs and the 90 % highest posterior densities (HPD) intervals of each parameter. We used the geometric mean of 2.49 × 10^−6^ of the mutation rates (per year) for both loci (1.33 × 10^−6^ for ITS and 4.69 × 10^−6^ for the *trnL-F*). The mutation rates were converted to per locus rate by multiplying by the fragment length in base pairs for conversion to demographic units, as required by IMa [88]. We used an 11-year generation time based on the observation that the age at maturity (seed production) begins ca. 2 years after seed germination and an assumed annual survivorship of 0.9 based on mistletoe seedling survivorship [30] to convert the effective populations size estimates. The approximate average generation time (T) is calculated according to T = *a* + [*s* ⁄ (1–*s*)] [90], where *a* is the time to maturity and *s* is the adult annual survival rate. Based on this, estimate for T was 11 years. To convert the IMa time since divergence parameter to years, *t*, we divided the time parameter (*B*) by the mutation rate per year (*U*) converted to per locus rate by multiplying by the fragment length in base pairs, and calculated for the mean rate described above.

### Evolutionary transition of mistletoe invasion

The evolutionary transition of mistletoe invasion was inferred from an approximate Bayesian computation in which evolutionary scenarios were simulated and compared through posterior probabilities [91] using the DIYABC software [92]. Populations covering the whole species’ distribution were analyzed to infer the history of the genetic structure indicated by phylogenetic and phylogeographic analyses of ITS and *trnL-F* sequences. Because three haplotype clusters are hypothesized to be associated with individuals/populations separated by habitat (BREE, SCHI, CALY; Additional file 2) with post-glacial population expansions and potentially corresponding to post-glacial invasion types (see Results), five evolutionary scenarios were built and tested considering haplotype network and *BEAST analyses: (a) three populations (Pop1, Pop2 and Pop3) have diverged simultaneously from an ancestral population at t1 (scenario 1, *null model*), which corresponded to the plant genetic groups, BREE, SCHI and CALY, respectively; (b) *isolation split model 1* (scenario 2), in which Pop1 (BREE) merged with Pop2 (SCHI) at t1 and subsequently with Pop3 (CALY) at t2; (c) *isolation split model 2* (scenario 3), in which Pop3 merged with Pop2 at t1, then both populations merged with Pop1 at t2; (d) *isolation split model 3* (scenario 4), in which Pop3 merged with Pop1 at t1 and then both populations merged with Pop2 at t2; and (e) *isolation with admixture model* (scenario 5), in which Pop2 was generated by admixture of Pops 1 and 3 at t1, then Pop1 merged with Pop3 at t2. Although there are numerous possible scenarios of divergence, we considered that these five scenarios represent the close relationships among the groups and the complex patterns of genetic divergence and admixture (see Results).

We generated 1 million simulated datasets per scenario considering a generalized stepwise-mutation model, uniform prior distributions for effective population sizes (10–100 000) and splitting events (100–50 000 generations). Comparison of scenarios was implemented in the DIYABC software [92]. The posterior probability of scenarios was assessed using a weighted polychotomous logistic regression on the 1 % of simulated datasets closest to the observed data [93]. For the best-supported scenario, we performed a model checking procedure by applying a PCA on test statistic vectors to visualize the fit between the simulated and the observed datasets. To assess the confidence in scenario choice, we simulated 500 pseudo-observed datasets (PODs) under each scenario to estimate Type I and Type II error rates [85]. Finally, point estimates for demographic and temporal parameters were obtained by local linear regression on the 1 % of simulations closest to the observed data set for the best-supported scenario [91, 92].

## Results

### Paleodistribution modeling and environmental variation

**Fig. 1:**
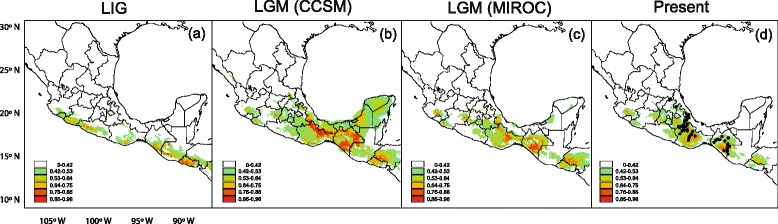
Results from the MAXENT analyses showing species distribution models for *Psittacanthus schiedeanus* (**a**) Last Inter Glacial (LIG, 140–120 ka), (**b**) Last Glacial Maximum (LGM, CCSM, 21 ka), (**c**) Last Glacial Maximum (LGM, MIROC, 21 ka), and (**d**) at present. The output of MAXENT consists of grid maps with each cell having an index of suitability between 0 and 1. Low values (*white to green*) indicate that the conditions are unsuitable for the species to occur, whereas high values (*orange to red*) indicate that the conditions are suitable. There is clear evidence that populations are connected during glacial cycles. Note that for maps b and c projections extend out into the ocean because of changes in sea levels

The SDM yielded a good fit for the current geographic distribution of *P. schiedeanus*. The AUC value for the replicate runs was 0.992 ± 0.003 (Fig. 1). Although there was some over prediction in certain geographic areas (the Trans-Mexican Volcanic Belt that was predicted by the model with moderate probability), the modeled distribution closely matches the current known distribution of *P. schiedeanus*. The paleodistribution modeling revealed that suitable habitat for *P. schiedeanus* during the LIG was more fragmented and restricted to mountain areas along the Pacific slope (Fig. 1a). However, the predictions for the LGM revealed that conditions of suitable habitat potentially expanded the distribution of *P. schiedeanus*, particularly in the eastern side of the Sierra Madre Oriental, Oaxaca and Chiapas applying both CCSM and MIROC simulations (Fig. 1b–c), and with a low probability into the Yucatan Peninsula under the CCSM scenario (Fig. 1b). Overall, the comparison between present and past climatic conditions predicted by the models suggests that climatic conditions of suitable habitat for *P. schiedeanus* experienced range shifts from the Pacific slope to east, expansion from LIG to LGM and contraction from LGM to present (Fig. 1a–d).

**Fig. 2:**
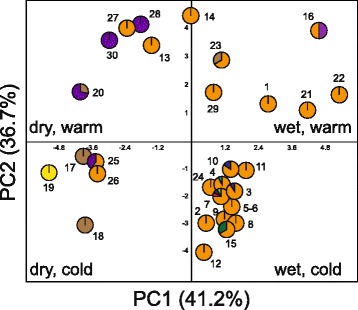
Principal components analysis (PCA) on the 19 BIOCLIM variables with the first principal component (PC1) largely a measure of precipitation conditions, and the second principal component (PC2) mainly determined by temperature measures. Numbers refer to collection sites (see Additional file 2) and pie charts represent *trnL-F* haplotypes found in each sampling locality (see Fig. 5)

The PCA of environmental data indicated two niche axes that together explained 77.9 % of the variation in *P. schiedeanus* (Fig. 2). The first niche axis (41.2 % of variation) was associated with precipitation variables (BIO12–BIO19), and mean temperature diurnal range (BIO2), isothermality (BIO3), temperature seasonality (BIO4) and temperature annual range (BIO7). The second niche axis (36.7 %) was associated with temperature variables (BIO1, BIO5–BIO6, BIO8–BIO11).

### Phylogenetic analysis, divergence time estimation and haplotype relationships

The aligned sequences of ITS region (*n* = 292) and the *trnL-F* intergenic spacer (*n* = 283) for *Psittacanthus* species were 569 and 321 base pairs (bp) long, respectively. Out of 1018 bp of the concatenated sequences, 588 sites were variable and out of these 464 were parsimony informative. In *P. schiedeanus* samples, we detected polymorphic sites in nine samples of the ITS region, with four of the 569 aligned sites (0.7 %). These were coded as ambiguous.

The trees obtained from the Bayesian analyses of the individual molecular markers (ITS and *trnL-F*) and the combined data set all differ in topology and degree of resolution. The *trnL-F* gene produced the poorest resolved tree (Additional file 4) and also contains the smallest number of nodes with posterior probability (PP) values above 0.90. The combined ITS and *trnL-F* data set produced the most resolved tree and contains the highest number of nodes with PP values above 0.90. The topology of the resulting BI tree showed monophyly of *Psittacanthus* samples (PP = 1; Additional file 4), with an early divergence of a clade composed of *P. ramiflorus*, *P. sonorae* and *P. palmeri*, followed by a clade with moderate support containing samples of *P. rhynchanthus* (PP = 1) and a clade with moderate support (PP = 0.86) containing samples of the Brazilian species (*P. robustus, P. biternatus, P. cordatus, P. acinarius*) and samples of *P. macrantherus, P. mayanus*, *P. auriculatus*, *P. calyculatus* and *P. schiedeanus*. Samples of *P. calyculatus* and samples of *P. schiedeanus* from the eastern Mexico cloud forests, central Oaxaca and Chiapas turned out to be monophyletic, but little support (PP = 0.67) and genetic structure was observed within this clade. The Bayes factor tests showed extensive incongruence between the chloroplast and the nuclear partitions. Combining ITS and *trnL-F* data sets produced a higher likelihood score than those for the alternative ITS or *trnL-F* data sets (2 × ln BF; ITS versus combined = 5031.742; *trnL-F* versus combined = 15946.436; ITS versus *trnL-F* = 10914.694).

**Fig. 3:**
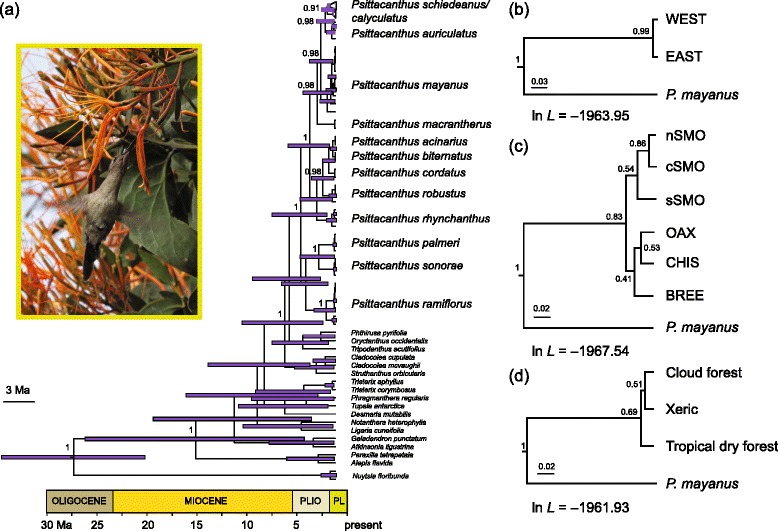
**a** Chronogram based on a Bayesian approach using a coalescent prior under an uncorrelated lognormal relaxed clock model and assuming constant population size of *Psittacanthus schiedeanus* sequences in BEAST. Pink bars indicate 95 % highest posterior density (HPD) intervals for nodes of particular interest. These nodes all have posterior probabilities above 0.9. **b** *BEAST model with simulation run with all samples as two lineages separated by the Isthmus of Tehuantepec (WEST and EAST), (**c**) samples as six lineages corresponding to geography and mountain ranges (nSMO, cSMO, sSMO, CHIS, OAX, BREE) or (**d**) three separate lineages according to habitat (*SCHI* cloud forests from San Luis Potosí to Oaxaca and Chiapas, *CALY* xeric vegetation in central Oaxaca, *BREE* tropical deciduous forests in Chiapas). Maximum-likelihood scores are indicated for *BEAST analyses (**b**–**d**). The likelihood scores under the three species delimitation hypothesis (SCHI, BREE, CALY) are compared with ln Bayes factors (BF) tests. The three-species hypothesis produced a higher likelihood score than those for the alternative hypotheses although the difference was not very strong (2 × ln BF; six-species hypothesis versus three-species hypothesis = 11.228; two-species hypothesis versus three-species hypothesis = 4.046; six-species hypothesis versus two-species hypothesis = 7.182)

The BEAST analyses placed the origin of the *Psittacanthus* crown clade in the Early Pliocene (Fig. 3). Although a split between *Psittacanthus* and a clade composed of samples of *Oryctanthus, Phthirusa, Tripodanthus, Cadocolea* and *Struthanthus* is recovered in the Bayesian analysis with strong support (PP = 1.0), the relationships within this clade were poorly resolved (Fig. 3). Within *Psittacanthus* the group formed by samples of *P. ramiflorus*, *P. palmeri* and *P. sonorae* split from other *Psittacanthus* species in the ingroup 5.41 MYA (95 % HPD 6.75–1.11 MYA) assuming constant population size. Samples of *P. schiedeanus* formed a well-supported monophyletic group (PP = 0.94), with diversification occurring in the early Pleistocene (2.55 MYA, 95 % HPD 1.57–0.23 MYA) and a split between *P. schiedeanus* and *P. auriculatus* estimated at 2.80 MYA (95 % HPD 2.08–0.31 MYA) assuming constant population size (Fig. 3).

**Fig. 4:**
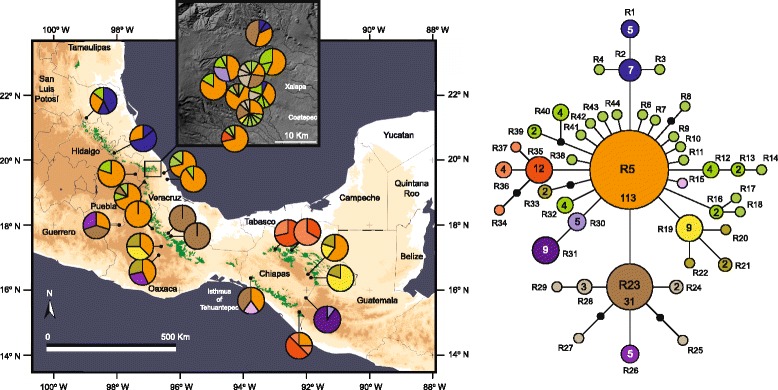
Geographic distribution and statistical parsimony network of 44 ITS ribotypes of *Psittacanthus schiedeanus*. Current natural range of cloud forests (indicated by *green shading*) is overlaid on a relief map of eastern Mexico. Pie charts represent ribotypes found in each sampling locality. Ribotype distributions at central Veracruz are shown separately in the inset

The aligned ITS data set for 251 samples of *P. schiedeanus* (559 bp) yielded 44 ribotypes (Additional file 5). In the ribotype network (Fig. 4), one ribotype (R5) was found in 113 of the 251 samples for 22 of the 29 sampled populations and forms the core of the network, from the northernmost population (Xilitla, San Luis Potosí) to Chiriquí, Panama. The most frequent and widespread ribotype (45 % of the individuals and 75.8 % of the sampled populations), inferred as ancestral based on its frequency and position in the network (Fig. 4), was separated by one or two mutations of most ribotypes in the network and they were singletons; it was not retrieved in two easternmost populations in Oaxaca and in several populations from Chiapas. The second most frequent and most widespread ribotype (R23) was found (31 samples) in three populations of Oaxaca (16 samples) and six populations from central Veracruz (13 samples) and one population from Chiapas (2 samples). Although ribotypes R1, R2, R19, R31, and R35 were not singletons they had certain geographic structure, with R1 and R2 mostly distributed in the northernmost populations (localities 1 and 2), R19 in two populations from Chiapas (5 samples), one from Veracruz (1 sample) and one population from Oaxaca (3 samples), R31 exclusively found in one population from Chiapas (9 samples), and R35 found in one population from Veracruz (1 sample) and three populations from Chiapas (11 samples).

**Fig. 5:**
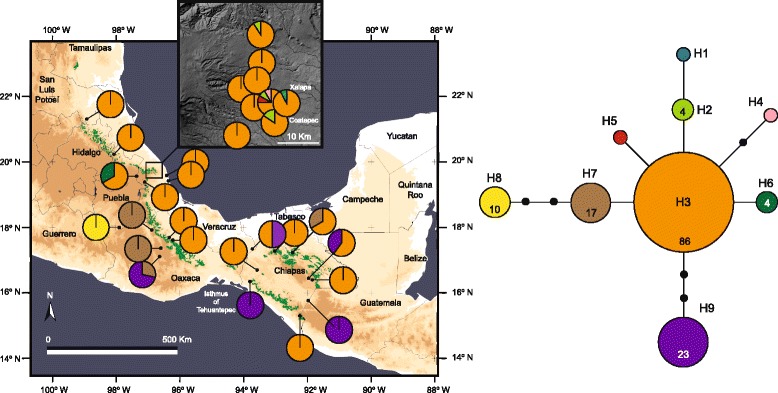
Geographic distribution and statistical parsimony network of 9 *trnL-F* haplotypes of *Psittacanthus schiedeanus*. Current natural range of cloud forests (indicated by green shading) is overlaid on a relief map of eastern Mexico. Pie charts represent haplotypes found in each sampling locality. Haplotype distributions at central Veracruz are shown separately in the inset

The aligned *trnL-F* data set for 242 samples of *P. schiedeanus* (282 bp) yielded 9 haplotypes (Fig. 5; Additional file 5). Statistical parsimony retrieved a well-resolved network, in which three main haplotypes could be distinguished (Fig. 5). Samples from cloud forest localities shared the most frequent (35.5 % of the individuals and 80.6 % of the sampled populations) haplotype (H3) forms the core of the “cloud forest” haplogroup, from Xilitla, San Luis Potosí to Santiago Comaltepec, Oaxaca and populations from Chiapas; it was not retrieved in the populations with more xeric vegetation in central Oaxaca or tropical deciduous forests in Chiapas. Haplotypes connected to H3 by one or more steps consists of haplotypes exclusively found in populations from central Veracruz (H1–H2, H4–H6), H7–H9 located in populations in central Oaxaca and H7 and H9 in populations from Chiapas with tropical deciduous forests (Fig. 5).

### Population genetic diversity indices, geographic structure and relationships among populations

Differentiation among populations based on ITS variation indicated that *P. schiedeanus* is genetically subdivided (*G*_ST_ = 0.295, SE = 0.0629). Genetic diversity across all populations (*h*_T_ = 0.804, SE = 0.0431; *v*_T_ = 0.807, SE = 0.1171) was higher than the average within-population (*h*_S_ = 0.567, SE = 0.0499; *v*_S_ = 0.484, SE = 0.0683). PERMUT analysis showed that *N*_ST_ (0.400, SE = 0.0639) was significantly higher (*p* <0.05) than that for *G*_ST_, indicating phylogeographical structuring. Similarly, phylogeographical structuring was observed in *P. schiedeanus* based on *trnL-F* variation (*G*_ST_ = 0.692, SE = 0.1058, *N*_ST_ = 0.741, SE = 0.1122; *N*_ST_ > *G*_ST_, *p* <0.05), with genetic diversity across all populations (*h*_T_ = 0.486, SE = 0.0925; *v*_T_ = 0.487, SE = 0.1221) much higher than the average within-population (*h*_S_ = 0.150, SE = 0.0497; *v*_S_ = 0.126, SE = 0.0533). Pairwise comparisons of *F*_ST_ values were not significant for ITS when groups of populations separated by habitat type were compared, whereas pairwise comparisons of *F*_ST_ values for *trnL-F* were high and significant (Table 1).

**Table 1:** Pairwise comparisons of *F*_ST_ values of ITS (above the diagonal) and *trnL-F* (below the diagonal) among populations of *Psittacanthus shiedeanus* grouped by habitat type

Habitat type	SCHI	BREE	CALY
SCHI	–	−0.0194	0.0176
BREE	**0.9686**	–	−0.0069
CALY	**0.9602**	**0.4827**	–

Significant values at *P* <0.001 are shown in bold. Habitat type abbreviations are as follows: *SCHI* cloud forests from San Luis Potosí to Oaxaca and Chiapas, *CALY* xeric vegetation in central Oaxaca, *BREE* tropical deciduous forests in Chiapas

The AMOVA results showed that 82.5 % of the genetic variation for ITS and 21.1 % for *trnL-F* was explained by differences within populations and 17.5 % and 78.9 %, respectively, by differences between populations when all locations were treated as a single group (Table 2). The AMOVA for the *trnL-F* revealed population structure, with highest *F*_CT_ value (*F*_CT_ = 0.78) obtained when populations are grouped by habitat type but differences between groups were not significant in the ITS data set (Table 2), suggesting that seed-mediated dispersal is more limited than pollen-mediated dispersal. When sampling sites are grouped as separated by the Isthmus of Tehuantepec or grouped by mountain range, significant but smaller proportion of the variation was attributed to differences between groups (Table 2). Nucleotide diversity (π) was low for both data sets (Additional file 6).

**Table 2:** Results of AMOVA models on *Psittacanthus schiedeanus* populations with no groups defined a priori (**a**), and grouped into east and west of the Isthmus of Tehuantepec (**b**), three groups according to habitat distribution (**c**) or grouped into six groups according to geography and mountain range (**d**)

			ITS					*trnL-F*		
	df	Sum of squares	Estimated variance	%	Fixation indices	df	Sum of squares	Estimated variance	%	Fixation indices
a. *No groups defined*										
Among populations	26	24.962	0.0689	17.53		26	130.07	0.55373	78.90	
Within populations	223	72.342	0.3244	82.47	*F*_ST_ = 0.17***	211	31.24	0.14808	21.10	*F*_ST_ = 0.78***
Total	249	97.304	0.3933			237	161.31	0.70181		
b. *Isthmus of Tehuantepec*										
Among groups	1	1.979	0.0163	4.00	*F*_CT_ = 0.04*	1	20.19	0.24972	28.47	*F*_CT_ = 0.28**
Among pop. within groups	25	23.103	0.0644	15.80	*F*_SC_ = 0.16***	25	109.45	0.47889	54.59	*F*_SC_ = 0.76***
Within populations	223	72.864	0.3267	80.20	*F*_ST_ = 0.19***	211	31.36	0.14865	16.94	*F*_ST_ = 0.83***
Total	249	97.946	0.4074			237	161.01	0.87726		
c. *Habitat type*										
Among groups	2	0.991	−0.0113	−2.93	*F*_CT_ = −0.02 ns	2	91.55	1.13235	78.31	*F*_CT_ = 0.78***
Among pop. within groups	24	23.971	0.0727	18.85	*F*_SC_ = 0.18***	24	38.52	0.16563	11.45	*F*_SC_ = 0.53***
Within populations	223	72.342	0.3244	84.08	*F*_ST_ = 0.15***	211	31.24	0.14808	10.24	*F*_ST_ = 0.89***
Total	249	97.304	0.3858			237	161.31	1.44605		
d. *Mountain range*										
Among groups	5	13.880	0.0739	17.49	*F*_CT_ = 0.17***	5	91.97	0.58369	63.63	*F*_CT_ = 0.64***
Among pop. within groups	21	11.202	0.0221	5.24	*F*_SC_ = 0.06*	21	37.68	0.18498	20.16	*F*_SC_ = 0.55***
Within populations	223	72.864	0.3267	77.27	*F*_ST_ = 0.23***	211	31.36	0.14865	16.21	*F*_ST_ = 0.84***
Total	249	97.946	0.4228			237	161.01	0.91732		

*ns* not significant (*P* >0.05), * *P* <0.01, ** *P* <0.001, *** *P* <0.0001

The *BEAST tree (Fig. 3b) of multilocus data for differentiation between clades separated by the Isthmus of Tehuantepec showed strong support (PP = 1; tree inset of Fig. 3), and the split occurred at ca. 0.098 MYA (95 % HPD: 0.16–0.047 MYA). However, this scenario was not the best supported compared with alternative species assignments (Fig. 3c–d).

### Demographic history

**Fig. 6:**
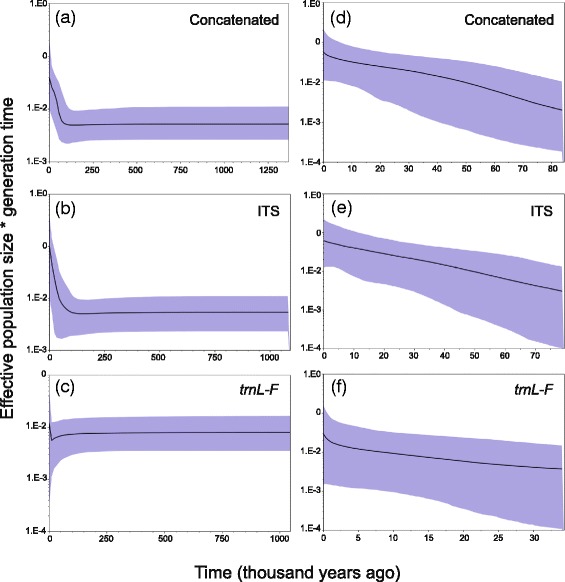
Bayesian skyline plots showing historical demographic trends of *Psittacanthus schiedeanus* for all pooled populations (**a–c**) and for the SCHI population (**d**–**f**) using the concatenated, ITS and *trnL-F* data sets, respectively. The *y* axis is the product between effective population size and the generation time and the *x* axis is time in thousands of years. Solid lines represent mean estimates and shaded areas represent 95 % confidence intervals. *SCHI* cloud forests from San Luis Potosí to Oaxaca and Chiapas

When populations are grouped as separated by habitat type, neutrality test values were negative and significant for SCHI (except Fu’s *Fs* in the *trnL-F*), indicating demographic expansion for SCHI (Additional file 6). In the mismatch distribution, sudden demographic expansion was not rejected for SCHI and CALY (Hri values) in the ITS, and in the *trnL-F* the Hri value was not rejected for SCHI (Additional file 6). The Bayesian skyline plots suggested that the effective population size increased in the SCHI population and the population as a whole around 80,000–10,000 years ago (Fig. 6). Changes in the effective population size were only observed in the concatenated and ITS data sets.

IMa results summarized in Table 3 are reported as the average of two runs of mean parameter estimates and the 95 % HPD intervals of each parameter. The ancestral population size (*N*_E_) is estimated to be higher than population sizes of the SCHI and CALY+BREE descendant populations (Table 3). Divergence time between the SCHI and the other populations separated by habitat type was estimated at ca. 1 MYA. When testing for migration during the time since population splitting, migration among populations occurred predominantly in one direction, moving from CALY+BREE to SCHI (Table 3).

**Table 3:** Results of isolation-with-migration model (IMa) for the split between groups of populations of *Psittacanthus schiedeanus*

	Model parameter estimates					
	*q*_*1*_	*q*_*2*_	*q*_*a*_	*t*	*M*_2-to-1_	*M*_1-to-2_
CALY+BREE vs. SCHI						
Mean	7.6121	3.4986	9.3720	2.735	0.0001	4.51
HPD95Lo	4.9616	1.9507	3.3077	0.955	0.0023	2.185
HPD95Hi	13.1038	6.8275	40.8594	9.45	0.0975	9.585
	Demographic parameter estimates					
	*Ne*_1_	*Ne*_2_	*Ne*_*a*_	*t*	*M*_2-to-1_	*M*_1-to-2_
CALY+BREE vs. SCHI						
Mean	69391.221	31892.318	85434.311	1097008	0.0004	7.8893
HPD95Lo	45229.956	17782.862	30153.152	383050	0.0058	2.1312
HPD95Hi	119453.521	62239.333	372470.625	3790395	0.6388	32.7210

Model parameters indicate estimates without use of molecular rate of evolution for six parameters (IMa output values). Demographic rates represent parameters scaled to rates of molecular evolution. Values are averages of two runs of mean parameter estimates and the 95 % highest posterior densities (HPD) intervals of each parameter: effective population sizes (*Ne*, individuals), migration rates (*Nm*, migrants per generation), estimated time since divergence (*t*, years). Population size (*Ne*) based on the average generation time (T) of 11 years for a high (0.9) annual adult survival rate. Habitat type abbreviations are as follows: *SCHI* cloud forests from San Luis Potosí to Oaxaca and Chiapas, *CALY* xeric vegetation in central Oaxaca, *BREE* tropical deciduous forests in Chiapas

### Evolutionary transition of mistletoe invasion

**Fig. 7:**
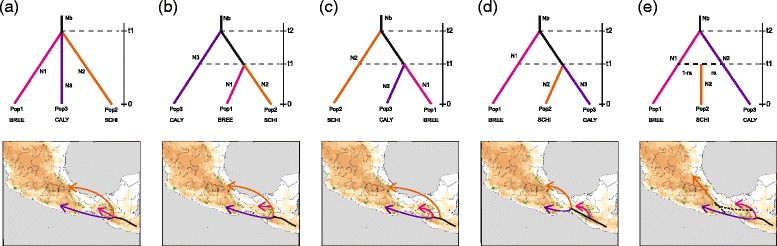
Competing demographic scenarios of *Psittacanthus schiedeanus* divergence and admixture. Because three haplotype clusters are hypothesized to be associated with individuals/populations separated by habitat (BREE, SCHI, CALY) with post-glacial population expansions and potentially corresponding to post-glacial invasion types (see Results), five evolutionary scenarios were defined and tested: (**a**) three populations (Pop1, Pop2 and Pop3) have diverged simultaneously from an ancestral population at t1 (scenario 1, *null model*), which corresponded to the plant genetic groups, BREE, SCHI and CALY, respectively; (**b**) *isolation split model 1* (scenario 2), in which Pop1 (BREE) merged with Pop2 (SCHI) at t1 and subsequently with Pop3 (CALY) at t2; (**c**) *isolation split model 2* (scenario 3), in which Pop3 merged with Pop2 at t1, then both populations merged with Pop1 at t2; (**d**) *isolation split model 3* (scenario 4), in which Pop3 merged with Pop1 at t1 and then both populations merged with Pop2 at t2; and (**e**) *isolation with admixture model* (scenario 5), in which Pop2 was generated by admixture of Pops 1 and 3 at t1, then Pop1 merged with Pop3 at t2. Comparison of the scenarios was implemented in the DIYABC software [92]. Habitat type abbreviations are as follows: *SCHI* cloud forests from San Luis Potosí to Oaxaca and Chiapas, *CALY* xeric vegetation in central Oaxaca, *BREE* tropical deciduous forests in Chiapas (Additional file 2)

Considering the five scenarios tested for nrDNA and cpDNA sequences, DIYABC analysis indicated that a simultaneous split (scenario 1) is the best-supported scenario (Fig. 7), with a posterior probability value much higher than those for the other scenarios. The 95 % confidence intervals for this model did not overlap with those obtained for the other scenarios (Table 4). Based on model performance, we were able to discriminate scenario 1 from other scenarios. This is corroborated by model checking, which showed a large cloud of data from the prior and observed datasets centered on a small cluster from the posterior predictive distribution, suggesting that the best supported scenario explained the observed data well (Results not shown). Analyses to estimate confidence in scenario choice based on 500 PODs indicate that Type I and Type II errors for the best-supported scenario were low (Table 4). Under scenario 1, posterior mean parameter estimates indicated that the simultaneous divergence occurred 21,780 generations ago. Assuming an 11-year generation time, the simultaneous split was dated to 217,800 years before present (Table 5), and probably pre-dates the Illinoian glacial stage (ca. 191,000–130,000 years ago). In accordance with the isolation and migration model, DIYABC estimated a smaller *Ne* for the descendant populations compared with that of the ancestral populations (Table 5). Estimated mean mutation rates of nrDNA and cpDNA were estimated to be 3.97 × 10^−8^ and 5.13 × 10^−8^, respectively (Table 5).

**Table 4:** Posterior probability of each scenario and 95 % confidence intervals (CI) based on the logistic regression approach for approximate Bayesian computation (ABC) analyses considering the groups of populations of CALY, BREE and SCHI mistletoes. Simulations and ABC analyses were performed considering both nrDNA and cpDNA sequences

Scenario	Posterior probability	95 % CI	Type I error	Type II error
1. Null model	0.6728	0.6565–0.6891	0.08	0.002
2. Isolation split model 1	0.0095	0.0000–0.0430		
3. Isolation split model 2	0.2586	0.2219–0.2954		
4. Isolation split model 3	0.0570	0.0258–0.0882		
5. Isolation with admixture model	0.0021	0.0000–0.0360		

Evolutionary scenarios are as follows: (1) *null model* (scenario 1) three populations (Pop1, Pop2 and Pop3) have diverged simultaneously from an ancestral population at t1, which corresponded to the plant genetic groups, BREE, SCHI and CALY, respectively; (2) *isolation split model 1* (scenario 2), in which Pop1 (BREE) merged with Pop2 (SCHI) at t1 and subsequently with Pop3 (CALY) at t2; (3) *isolation split model 2* (scenario 3), in which Pop3 merged with Pop2 at t1, then both populations merged with Pop1 at t2; (4) *isolation split model 3* (scenario 4), in which Pop3 merged with Pop1 at t1 and then both populations merged with Pop2 at t2; and (5) *isolation with admixture model* (scenario 5), in which Pop2 was generated by admixture of Pops 1 and 3 at t1, then Pop1 merged with Pop3 at t2. Habitat type abbreviations are as follows: *SCHI* cloud forests from San Luis Potosí to Oaxaca and Chiapas, *CALY* xeric vegetation in central Oaxaca, *BREE* tropical deciduous forests in Chiapas

**Table 5:** Posterior parameter estimates (median and 95 % confidence intervals) for the best-supported scenario (scenario 1) considering the three mistletoe groups (BREE, SCHI and CALY). Estimates are based on 1 % of simulated datasets closest to the observed values. Simulations and approximate Bayesian computation (ABC) analyses were performed considering both nrDNA and cpDNA sequences

Parameter	Median	q[2.5]	q[97.5]
N1 (BREE)	1.14e + 04	2.08e + 03	4.39e + 04
N2 (SCHI)	1.71e + 05	1.12e + 05	1.98e + 05
N3 (CALY)	2.78e + 04	7.45e + 03	1.21e + 05
t1	1.98e + 04	5.88e + 03	3.80e + 04
Na	1.33e + 05	2.98e + 04	4.29e + 05
*μ*_nrDNA_	3.97e-08	1.98e-08	7.77e-08
*μ*_cpDNA_	5.13e-08	2.14e-08	9.33e-08

Parameters are *N* = effective population size for BREE (pop1, N1), SCHI (Pop2, N2), CALY (Pop3, N3), and the ancestral populations (Na), *t* = time since divergence after the Last Glacial Maximum (t1) and during Last Interglacial (t2), and *μ* = mutation rate for nuclear and chloroplast DNA, respectively. Habitat type abbreviations are as follows: *SCHI* cloud forests from San Luis Potosí to Oaxaca and Chiapas, *CALY* xeric vegetation in central Oaxaca, *BREE* tropical deciduous forests in Chiapas

## Discussion

### Mistletoes in the Mesoamerican cloud forests

In northern Mesoamerica, cloud forests encompass an extremely heterogeneous mixture of North American temperate tree species that are present in the region since the Tertiary and tropical species with South American origins during the early Miocene [39–42]. The dynamic Mesoamerican geological landscape from the Miocene to the Pliocene and the repeated cycles of cloud forest contraction and expansion due to Pleistocene climatic conditions [94, 95] affected the distribution and composition of cloud forests in the region, and contributed to both ancient [32, 46, 96, 97] and more recent divergences producing a common phylogeographic break at the Isthmus of Tehuantepec (e.g., [32, 34, 98]). The emerging phylogeographical patterns of temperate tree species that have migrated south from North America have been attributed to isolation by arid conditions during the Pliocene and climate changes during Pleistocene glaciations that promoted the expansion, contraction and divergence of populations [46, 99]. However, plant species that presumably colonized northern Mesoamerica from South America are little studied.

Seven studies have explored the phylogeography of widespread, cloud forest-adapted plant species across northern Mesoamerica. High levels of genetic variation and geographical structuring were inferred for wind-dispersed *Alsophila firma* (Baker) D.S. Conant (Cyatheaceae) [58] and *Liquidambar styraciflua* L. (Altingiaceae) [46] or bird-dispersed *Podocarpus matudae* Lundell (Podocarpaceae), *Palicourea padifolia* (Humb. & Bonpl. ex Schult.) C.M. Taylor & Lorence (Rubiaceae) and *Rhipsalis baccifera* (Sol.) Stearn (Cactaceae) [34, 98, 100]. On the other hand, for wind/gravity dispersed *Begonia heracleifolia* Cham. & Schltdl. (Begoniaceae) [101] and *Moussonia deppeana* (Schltdl. & Cham.) Hanst. (Gesneriaceae) [33], the genetic variation and genetic structure were inferred to be stronger. The high genetic differentiation of populations with strong spatial structuring provides support for the persistence of populations in situ during dry periods in the Pleistocene, in which populations remained isolated within the current fragmented distribution of the cloud forest [33, 101], suggesting that the vegetation along the Isthmus of Tehuantepec has acted as a barrier to seed dispersal and that the multiple vicariance events observed in various taxa occurred at earlier different times [32, 34, 98]. However, the widely documented genetic break at the Isthmus of Tehuantepec in several taxa [32, 96], though significant for the plastid *trnL-F* data, only explained 28 % of the total variation according to the AMOVA model, suggesting that low levels of seed flow have occurred across this barrier.

We found population genetic structure in *P. schiedeanus*, with differentiation between groups of populations separated by habitat type. Despite the genetic differentiation of some *P. schiedeanus* populations, there are widespread ribotypes and haplotypes in the ITS and *trnL-F* markers, respectively. In particular, the lower variation found in ITS and the presence of widespread ribotypes in the region are suggestive of effective nuclear gene flow via pollen (e.g., [34, 102]). In contrast, the population differentiation of *P. schiedeanus* shown by the plastid *trnL-F* data suggests that seed flow has been more restricted in some cases. Our results from the pairwise *F*_ST_ comparisons and AMOVA analysis suggest fairly high levels of genetic diversity among populations and habitat types of *P. schiedeanus*, especially when considering the cpDNA. However, significant evidence of population expansions was found in cpDNA sequences and the most frequent haplotypes were retrieved in most cloud forest areas of *P. schiedeanus*. BSPs, negative values of Tajima’s *D*, Fu’s *Fs* and the results of mismatch distribution for the plastid marker suggest that the population in the cloud forests (SCHI) has experienced recent demographic expansion, most likely during the LGM.

Predictions of SDMs under past LGM climatic conditions and the significant signal of demographic expansion suggest that *P. schiedeanus* (SCHI) populations experienced a range expansion tracking the conditions of the cloud forest distribution and shifted to the lowlands with population connectivity during the LGM, as predicted by the moist forests model [57, 58]. In addition, the Bayesian skyline plot for the SCHI population detected a significant increase in the overall effective population size dating to sometime during the LGM, when sudden demographic expansion occurred and descent to lower elevations allowed populations to gene flow. However, patterns of high genetic diversity among different populations do not fully support this hypothesis. The distribution of haplotype diversity suggests that the low genetic structure observed among populations of cloud forest areas is due to population connectivity across low-elevation barriers. Accordingly, we would hypothesize that during glacial periods, populations of *P. schiedeanus* expanded to the lowlands, gene flow was extensive, and habitats in which populations had gone extinct during the interglacials were probably re-colonized. Throughout interglacial periods, populations fragmented and contracted as they moved up in elevation with the warming climate and genetic divergence commenced across cloud forest areas dissected by mountains and low-elevation barriers.

Despite the appeal of the contraction/expansion model of populations of cloud forest-adapted species throughout glacial-interglacial climate cycles by shifting elevation, there is little evidence for a common demographic pattern of co-distributed species [57]. Ruiz-Sanchez and Ornelas [46] analyzed the phylogeographical structure of *Liquidambar styraciflua* (Altingiaceae), a deciduous tree commonly parasitized by *P. schiedeanus* with a disjunct distribution between the deciduous forest of the southeastern USA and the Mesoamerican cloud forest, and found signals of demographic expansion of Mesoamerican populations and habitat suitability connected and expanded to lower coastal areas under LGM climatic conditions. However, these patterns of genetic structure are not shared by *P. schiedeanus*. The observed inconsistency between demographic histories of *P. schiedeanus* and its most frequent host tree species in cloud forests can be the result of a differential response of species to climate change.

### The role of historical climate changes on mistletoe evolution

The phylogeographical structure found in *P. schiedeanus* suggests that three lineages occupy geographic regions separated by habitat type, driving isolation and genetic divergence between groups of populations enhanced by environmental differences. Despite the high levels of genetic diversity and genetic structure of cpDNA among groups of populations (significant divergence in *F*_ST_ values and AMOVA), the occurrence of widespread plastid haplotypes in most areas (i.e., post-glacial population expansion) of the range of *P. schiedeanus* is consistent with the hypothesis of effective seed dispersal episodes. Accordingly, the mobility of bird dispersal agents has erased the genetic structure predicted to groups of populations along the cloud forest distribution (e.g., [32, 34]. It is possible that large-scale seed recruitment patterns produced by frugivorous birds that consume and eventually disperse mistletoe seeds might have led to increased gene flow among cloud forest areas.

Several bird species consuming fruits of *P. schiedeanus* mistletoes have been reported, the most important being cedar waxwings (*Bombycilla cedrorum* Vieillot, Bombycillidae), grey silky-flycatchers (*Ptilogonys cinereus* Swainson, Ptilogonatidae), and social flycatchers (*Myiozetetes similis* Spix, Tyrannidae) [14, 20, 36, 37]. The foraging and flocking behavior and local abundance bird dispersers differ widely [14, 36], and, consequently, affect differently the spatial patterns of mistletoe seed deposition [14]. On their winter migration cedar waxwings usually arrive in groups of 15–80 individuals on a single infected tree, whereas grey silky-flycatchers and social flycatchers occur in pairs or small flocks of 3–6 individuals. The winter migration route of cedar waxwings overlaps the distribution of *P. schiedeanus*, and the timing of its arrival coincides with the mistletoe fruiting season [36]. Bird migration could produce a large-scale seed rain pattern in a north to south direction, and this current directional gene flow could have erased past phylogeographic patterns.

Because cedar waxwings and grey silky-flycatchers tend to perch on mistletoe infected trees, their residence time on the most abundant host species ranges from 9 to 30 min [14] and defecate *P. schiedeanus* seeds within a short time of ingestion (ca. 20–30 min; [20]), it is also possible that the dispersal of *P. schiedeanus* may be geographically limited, leading to its local adaptation restricted to available host trees along cloud forests. Cedar waxwings defecate more mistletoe seeds onto branches of *L. styraciflua* individuals than onto those of other tree species [14], and more seedlings survive on branches of the most frequently infected host species, *L. styraciflua* [30]. In contrast with other host tree species, the higher germination rates of bird gut-processed mistletoes collected on *Liquidambar* trees is consistent with the prevalence and spatial distribution patterns reported by previous studies [14, 30, 36], and cross-infection experiments have shown the potential for local host adaptation in *P. schiedeanus* [20]. In this way, depending on the heterogeneity of host patches at a local scale, some mistletoe plants can develop more specificity on particular host trees that may lead to gene flow changes and the eventual formation of mistletoe races [5, 15, 18, 20]. However, the large host range suggests that *P. schiedeanus* is not host-specific, and in areas where *Liquidambar* is absent the parasite-host interaction is predominantly with other host species. Thus, geologic and climate-driven processes implicated in the fragmentation of the Mesoamerican cloud forests and, consequently the distribution of potential host species at a larger geographical scale, as well as selection pressures imposed by floral visitors and seed dispersers, could have influenced the distribution of the genetic variation among populations of *P. schiedeanus*. If the historical fragmentation of cloud forest patches or selection reduced gene flow among *P. schiedeanus* populations, host specificity may lead to genetic divergence and ultimately allopatric speciation.

However, the ABC analyses strongly supported the scenario of simultaneous divergence among the three groups dating back ca. 200 k years ago. This divergence time estimate for the groups of populations could have resulted from the low power of ABC analyses and our set of nuclear and chloroplast DNA sequences to detect subtle genetic differences in divergence times among groups of populations that have been isolated more recently. Under this scenario, climatic fluctuations throughout the Pleistocene would have altered the distribution of suitable habitat for mistletoes throughout Mesoamerica leading to variation in population continuity and isolation. Species that occur in topographically complex regions will frequently move between high and low elevation habitats in response to climatic fluctuations during glacial cycles [57]. Furthermore, the plant density and the clumped spatial distribution of conspecifics may affect hummingbird foraging behavior. Hummingbirds may forage more locally in search of nectar in high-density populations of *P. schiedeanus*, thereby reducing the dispersal distances of pollen. Therefore, pollen dispersal distance is expected to be lower in a high-density species distributed in a naturally fragmented habitat where individuals are locally aggregated on cloud forest host trees. However, its wide host and geographical ranges and large-scale seed recruitment patterns produced by long-distance bird migrants that consume and eventually disperse mistletoe seeds might have lead to increased gene flow, thus obscuring the geographical genetic structure created by cloud forest isolation before and during the Pleistocene.

## Conclusions

Our phylogeographic approach allowed us to clarify relationships among populations of *P. schiedeanus* mainly distributed among cloud forests (SCHI) and the distribution boundaries with phenotypically similar *Psittacanthus* populations allopatrically distributed in more xeric environments (BREE in tropical dry forests of the Central Depression in Chiapas and CALY in more xeric plant communities of central Oaxaca). Genetic differentiation among these groups was strong and significant for the plastid *trnL-F* marker and non-significant for the nuclear ITS. Collectively, our data suggest that geographical, ecological, historical and environmental factors are the main drivers of genetic differentiation of these Mesoamerican parrot-flower mistletoes. According to SDMs, population expansion tests, skyline plots and divergence time estimations, the invasion of *P. schiedeanus* to cloud forests in eastern Mexico is relatively recent, and the invasion occurred to host tree species with longer histories in the region (e.g., [46]). Nonetheless, to better understand and manage threats of mistletoe populations, including potential transmission pathways of these commercially and ecologically important pathogens [1, 4] and genetic dilution from hybridization between *P. schiedeanus* and other sympatric *Psittacanthus* species, a more detailed sampling within the distribution of closely related *P. calyculatus* and *P. breedlovei* along the Trans-Mexican Volcanic Belt and tropical dry forests in the Central Depression of Chiapas is needed. Also, the genotyping of individuals using specifically designed microsatellites [103] will be critically important to the understanding of *Psittacanthus* species boundaries.

## Declarations

### Ethics approval

No aspect of this study required ethics approval.

### Consent for publication

No aspect of this study required written informed consent to participate.

### Availability of data and materials

Sequence reads can be accessed through GenBank under the Accession Numbers KU922961–KU923047 (ITS), *trnL-F*: KU923270–KU923321 (*trnL-F*). The data sets (alignments of ITS and *trnL-F* and IMa and MAXENT input files) supporting the results of this article are available in the Dryad Digital Repository (http://dx.doi.org/10.5061/dryad.t49h6) as Ornelas et al. [104].

## Additional files

Additional file 1: Map showing collecting sites of *Psittacanthus schiedeanus*. Numbers refer to collection sites and thin ellipses show collecting sites within the cloud forest areas. Region abbreviations are as follows: *nSMO* northern Sierra Madre Oriental, *cSMO* central Sierra Madre Oriental, *sSMO* southern Sierra Madre Oriental, *CHIS* Chiapan Highlands (cCHIS and pCHIS) separated by the Central Depression that together with Guatemala form the region Trans-Isthmian Highlands (TIH), *OAX* Oaxacan drylands. (PDF 5098 kb) Additional file 2: Taxon sampling. Voucher information, geographic location and population code of the 31 *Psittacanthus schiedeanus* populations used in the study. IDs reported refer to accession numbers in the Instituto de Ecología, AC (XAL) herbarium. (DOC 150 kb) Additional file 3: Outgroup sampling. Geographic location, GenBank accession numbers and voucher information of *Psittacanthus* species used in this study. IDs reported refer to accession numbers in the Instituto de Ecología, AC (XAL) and Universidade de São Pablo (USP) herbaria. (DOC 83 kb) Additional file 4: Phylogenetic trees of ITS, *trnL-F* and ITS+*trnL-F*. Bayesian posterior probabilities for MrBayes analyses. Illustrations of tree topologies based on (a) ITS, (b) *trnL-F*, and (c) concatenated sequences for samples of the *Psittacanthus* clade. The values above branches denote posterior probabilities (PP). The distal portion of the phylogeny based on concatenated sequences is shown, representing the *Psittacanthus schiedeanus* samples through other *Psittacanthus* species (d). (PDF 325 kb) Additional file 5: Haplotype information. Number of genetically analyzed samples (*n*) for each molecular marker (ITS and *trnL-F*) and number of distinct ribotypes (R) and haplotypes (H) found in *Psittacanthus schiedeanus* individuals sampled, and the number of individuals per ribotype or haplotype in parentheses. Codes are from networks in Figs. 4 and 5. (DOC 73 kb) Additional file 6: Summary statistics. Summary statistics of demographic analysis of *Psittacanthus schiedeanus* samples by habitat type to infer demographic range expansion. (DOC 39 kb)

## Abbreviations

ABC: approximate Bayesian computation
AMOVAs: analyses of molecular variance
AUC: area under the receiver operating characteristic curve
BI: Bayesian inference
bp: base pairs
CCSM: community climate system model
ESS: effective sample size
HPD: highest posterior probabilities
Hri: Harpending’s raggedness index
IM: isolation with migration
LGM: last glacial maximum
LIG: last inter glacial
MCMC: Markov Chain Monte Carlo
MIROC: model for interdisciplinary research on climate
MYA: million years ago
PCA: principal components analysis
PODs: pseudo-observed datasets
PP: posterior probabilities
SDM: species distribution modeling
SSD: sum of squares differences
TMVP: Trans-Mexican Volcanic Belt

## References

[1] Mathiasen RL, Nickrent DL, Shaw DC, Watson DM (2008). Mistletoes: pathology, systematics, ecology, and management. Plant Dis.

[2] Kuijt J (1969). The biology of parasitic flowering plants.

[3] Kuijt J (2009). Monograph of *Psittacanthus* (Loranthaceae). Systematic Botany Monographs. American Society of Plant Taxonomists.

[4] Watson DM (2001). Mistletoe –a keystone resource in forests and woodlands worldwide. Annu Rev Ecol Syst.

[5] Norton DA, Carpenter MA (1998). Mistletoes as parasites: host specificity and speciation. Trends Ecol Evol.

[6] Amico GC, Vidal-Russell R, Garcia MA, Nickrent DL (2012). Evolutionary history of the South American mistletoe *Tripodanthus* (Loranthaceae) using nuclear and plastid markers. Syst Bot.

[7] Lira-Noriega A, Toro-Núñez O, Oaks JR, Mort ME (2015). The roles of history and ecology in chloroplast phylogeographic patterns of the bird-dispersed plant parasite *Phoradendron californicum* (Viscaceae) in the Sonoran Desert. Am J Bot.

[8] Price PW (1980). Evolutionary biology of parasites.

[9] Kuijt J (2014). Five new species, one new name, and transfers in Neotropical mistletoes (Loranthaceae), miscellaneous notes, 61–68. Novon.

[10] Barlow BA, Wiens D (1977). Host-parasite resemblance in Australian mistletoes: the case for cryptic mimicry. Evolution.

[11] Howell BE, Kenaley S, Mathiasen R (2006). First report of *Psittacanthus macrantherus* on *Pinus devoniana* and *Quercus castanea* in Mexico. Plant Dis.

[12] Mathiasen RL, Daugherty CM, Howell BE, Melgar JC, Sesnie SE (2007). New morphological measurements of *Psittacanthus angustifolius* and *Psittacanthus pinicola* (Loranthaceae). Madrono.

[13] Pérez Crespo MJ, Ornelas JF, Martén-Rodríguez S, González-Rodríguez A, Lara C (2016). Reproductive biology and nectar production of the Mexican endemic *Psittacanthus auriculatus* (Loranthaceae), a hummingbird-pollinated mistletoe. Plant Biol.

[14] López de Buen L, Ornelas JF (1999). Frugivorous birds, host selection and the mistletoe *Psittacanthus schiedeanus*, in central Veracruz, Mexico. J Trop Ecol.

[15] Lara C, Pérez G, Ornelas JF (2009). Provenance, guts, and fate: field and experimental evidence in a host-mistletoe-bird system. Ecoscience.

[16] May DS (1971). The role of population differentiation in experimental infection of *Prosopis* by *Phoradendron*. Am J Bot.

[17] Clay K, Demet D, Rejmanek M (1985). Experimental evidence for host races in mistletoe *Phoradendron tomentosum* (Viscaceae). Am J Bot.

[18] Overton JM (1997). Host specialization and partial reproductive isolation in desert mistletoe (*Phoradendron californicum*). Southwest Nat.

[19] Norton DA, Ladley J, Sparrow AD (2002). Host provenance effects on germination and establishment of two New Zealand mistletoes (Loranthaceae). Funct Ecol.

[20] Ramírez MM, Ornelas JF (2012). Cross-infection experiments of *Psittacanthus schiedeanus*: effects of host provenance, gut passage and host fate on mistletoe seedling survival. Plant Dis.

[21] Okubamichael DY, Griffiths ME, Ward D (2014). Reciprocal transplant experiment suggests host specificity of the mistletoe *Agelanthus natalitius* in South Africa. J Trop Ecol.

[22] Glazner JT, Devlin B, Ellstrand N (1988). Biochemical and morphological evidence for host race evolution in desert mistletoe, *Phoradendron californicum* (Viscaceae). Plant Syst Evol.

[23] Nickrent DL, Butler TL (1990). Allozimic relationships of *Arceuthobium campylopodum* and allies in California. Biochem Syst Ecol.

[24] Nickrent DL, Butler TL (1991). Genetic relationships in *Arceuthobium monticola* and *A. siskiyouense* (Viscaceae): New dwarf mistletoe species from California and Oregon. Biochem Syst Ecol.

[25] Nickrent DL, Stell AL (1990). Electrophoretic evidence for genetic differentiation in two host races of hemlock dwarf mistletoe (*Arceuthobium tsugense*). Biochem Syst Ecol.

[26] Linhart YB, Malville Ellwood L, Karron JD, Gehring JL (2003). Genetic differentiation in the dwarf mistletoes *Arceuthobium vaginatum* and *Arceuthobium americanum* on their principal and secondary hosts. Int J Plant Sci.

[27] Jerome CA, Ford BA (2002). The discovery of three genetic races of the dwarf mistletoe *Arceuthobium americanum* (Viscaceae) provides insight into the evolution of parasitic angiosperms. Mol Ecol.

[28] Amico GC, Nickrent DL (2009). Population structure and phylogeography of the mistletoes *Tristerix corymbosus* and *T. aphyllus* (Loranthaceae) using chloroplast DNA sequence variation. Am J Bot.

[29] Zuber D, Widmer A (2009). Phylogeography and host race differentiation in the European mistletoe (*Viscum album* L.). Mol Ecol.

[30] López de Buen L, Ornelas JF (2002). Host compatibility of the cloud forest mistletoe *Psittacanthus schiedeanus* (Loranthaceae) in central Veracruz, Mexico. Am J Bot.

[31] Rödl T, Ward D (2002). Host recognition in a desert mistletoe: early stages of development are influenced by substrate and host origin. Funct Ecol.

[32] Ornelas JF, Sosa V, Soltis DE, Daza JM, González C, Soltis PS, Gutiérrez-Rodríguez C, Espinosa de los Monteros A, Castoe TA, Bell C, Ruiz-Sanchez E . Ruiz-Sanchez E. Comparative phylogeographic analyses illustrate the complex evolutionary history of threatened cloud forests of northern Mesoamerica. PLoS ONE. 2013;8:e56283. 10.1371/journal.pone.0056283 PMC3567015 23409165

[33] Ornelas JF, González C (2014). Interglacial genetic diversification of *Moussonia deppeana* (Gesneriaceae), a hummingbird-pollinated, cloud forest shrub in northern Mesoamerica. Mol Ecol.

[34] Ornelas JF, Rodríguez-Gómez F (2015). Influence of Pleistocene glacial/interglacial cycles of the genetic structure of the mistletoe cactus *Rhipsalis baccifera* (Cactaceae) in Mesoamerica. J Hered.

[35] Ramírez MM, Ornelas JF (2010). Pollination and nectar production of *Psittacanthus schiedeanus* (Loranthaceae) in central, Veracruz, Mexico. Bol Soc Bot Méx.

[36] Ramírez MM, Ornelas JF (2009). Germination of *Psittacanthus schiedeanus* (mistletoe) seeds after passage through the gut of Cedar Waxwings and Grey Silky-Flycatchers. J Torrey Bot Soc.

[37] López de Buen L, Ornelas JF (2001). Seed dispersal of the mistletoe *Psittacanthus schiedeanus* by birds in central Veracruz, Mexico. Biotropica.

[38] Sánchez-González LA, Navarro-Sigüenza AG, Ornelas JF, Morrone JJ (2013). What’s in a name? Mesoamerica. Rev Mex Biodivers.

[39] Miranda F, Sharp AJ (1950). Characteristics of the vegetation in certain temperate regions of eastern Mexico. Ecology.

[40] Sharp AJ (1951). The relation of the Eocene Wilcox flora to some modern floras. Evolution.

[41] Williams-Linera G (1997). Phenology of deciduous and broadleaved-evergreen tree species in a Mexican tropical lower montane forest. Glob Ecol Biogeogr Lett.

[42] Graham A (1999). Studies in Neotropical paleobotany. XIII. An Oligo-Miocene palynoflora from Simojovel (Chiapas, Mexico). Am J Bot.

[43] Burger W, Kuijt J, Burger W (1983). Loranthaceae *sensu lato*. Flora Costarricensis. Feldiana Botany, New Series 13, Publication 1350.

[44] Cházaro M, Oliva R (1988). Loranthaceae del centro de Veracruz y zona limítrofe de Puebla, IV. Cact Suc Mex.

[45] López de Buen L, Ornelas JF, García-Franco JG (2002). Mistletoe infection of trees located at fragmented forest edges in the cloud forests of central Veracruz, Mexico. For Ecol Manag.

[46] Ruiz-Sanchez E, Ornelas JF (2014). Phylogeography of *Liquidambar styraciflua* (Altingiaceae) in Mesoamerica: survivors of a Neogene widespread temperate forest (or cloud forest) in North America?. Ecol Evol.

[47] Azpeitia F, Lara C (2006). Reproductive biology and pollination of the parasitic plant *Psittacanthus calyculatus* (Loranthaceae) in central Mexico. J Torrey Bot Soc.

[48] Howell BE, Mathiasen RL (2004). Growth impacts of *Psittacanthus angustifolius* Kuijt on *Pinus oocarpa* Schiede in Honduras. For Ecol Manag.

[49] Wilson CA, Calvin CL (2006). An origin of aerial branch parasitism in the mistletoe family, Loranthaceae. Am J Bot.

[50] Amico GC, Vidal-Russell R, Nickrent DL (2007). Phylogenetic relationships and ecological speciation in the mistletoe *Tristerix* (Loranthaceae): the influence of pollinators, dispersers, and hosts. Am J Bot.

[51] Vidal-Russell R, Nickrent DL (2008). Evolutionary relationships in the showy mistletoe family (Loranthaceae). Am J Bot.

[52] Doyle JJ, Doyle JL (1987). A rapid DNA isolation procedure from small quantities of fresh leaf tissue. Phytochem Bull.

[53] Suh Y, Thien LB, Reeve HE, Zimmer EA (1993). Molecular evolution and phylogenetic implications of internal transcribed spacer sequences of ribosomal DNA in Winteraceae. Am J Bot.

[54] White TJ, Burns T, Lee S, Taylor J (1990). Amplification and direct sequencing of fungal ribosomal RNA genes for phylogenetics. PCR protocols: a guide to methods and applications.

[55] Taberlet P, Gielly L, Pautou G, Bouvet J (1991). Universal primers for amplification of three non-coding regions of chloroplast DNA. Plant Mol Biol.

[56] Elith J, Phillips SJ, Hastie T, Dudík M, Chee YE, Yates CJ (2011). A statistical explanation of MaxEnt for ecologists. Divers Distrib.

[57] Ramírez-Barahona S, Eguiarte LE (2013). The role of glacial cycles in promoting genetic diversity in the Neotropics: the case of cloud forests during the Last Glacial Maximum. Ecol Evol.

[58] Ramírez-Barahona S, Eguiarte LE (2014). Changes in the distribution of cloud forests during the last glacial period predict the patterns of genetic diversity and demographic history of the tree fern *Alsophila firma* (Cyatheaceae). J Biogeogr.

[59] Phillips SJ, Anderson RP, Schapire RE (2006). Maximum entropy modeling of species geographic distributions. Ecol Model.

[60] Hijmans RJ, Cameron SE, Parra JL, Jones PG, Jarvis A (2005). Very high resolution interpolated climate surfaces fro global land areas. Int J Climatol.

[61] Braconnot P, Otto-Bliesner B, Harrison S, Joussaume S, Peterschmitt JY, Abe-Ouchi A, et al. Results of PMIP2 coupled simulations of the Mid-Holocene and Last Glacial Maximum –Part 2: feedbacks with emphasis on the location of the ITCZ and mid- and high latitudes heat budget. Clim Past. 2007;3(2):279–96.

[62] Collins WD, Bitz CM, Blackmon ML, Bonan GB, Bretherton CS, Carton JA, et al. The community climate system model: CCSM3. J Clim. 2004;19(11):2122–43.

[63] Hasumi H, Emori S (2004). K-1 coupled GCM (MIROC) description.

[64] Otto-Bliesner BL, Marshall SJ, Overpeck JT, Miller GH, Hu A (2006). Simulating Arctic climate warmth and icefield retreat in the Last Interglaciation. Science.

[65] Otto-Bliesner BL, Hewitt CD, Marchitto TM, Brady E, Abe-Ouchi A, Crucifix M, et al. Last Glacial Maximum ocean thermohaline circulation: PMIP2 model intercomparisons and data constraints. Geophys Res Lett. 2007;34(12):L12706.

[66] Poelchau MF, Hamrick JL (2011). Palaeodistribution modelling does not support disjunct Pleistocene refugia in several Central American plant taxa. J Biogeogr.

[67] Huelsenbeck JP, Ronquist F (2001). MRBAYES: Bayesian inference of phylogeny. Bioinformatics.

[68] Ronquist F, Huelsenbeck J (2003). MrBayes 3: Bayesian phylogenetic inference under mixed models. Bioinformatics.

[69] Miller MA, Pfeiffer W, Schwartz T (2010). Creating the CIPRES Science Gateway for inference of large phylogenetic trees.

[70] Posada D (2008). jModelTest: phylogenetic model averaging. Mol Biol Evol.

[71] Drummond AJ, Rambaut A (2007). BEAST: Bayesian evolutionary analysis by sampling trees. BMC Evol Biol.

[72] Vidal-Russell R, Nickrent DL (2008). The first mistletoes: origins of aerial parasitism in Santalales. Mol Phylogenet Evol.

[73] Kay KM, Whittall JB, Hodges SA (2006). A survey of nuclear ribosomal internal transcribed spacer substitution rates across angiosperms: an approximate molecular clock with life history effects. BMC Evol Biol.

[74] Richardson JE, Pennington RT, Pennington TD, Hollingsworth PM (2001). Rapid differentiation of a species-rich genus of Neotropical rain forest trees. Science.

[75] Clement M, Posada D, Crandall KA (2000). TCS: a computer program to estimate gene genealogies. Mol Ecol.

[76] Pfenninger M, Posada D (2002). Phylogeographic history of the land snail *Candidula unifasciata* (Helicellinae, Stylommatophora): Fragmentation, corridor migration, and secondary contact. Evolution.

[77] Pons O, Petit RJ (1996). Measuring and testing genetic differentiation with ordered versus unordered alleles. Genetics.

[78] Excoffier L, Laval G, Schneider S (2005). Arlequin ver. 3.0: an integrated software package for population genetics data analysis. Evol Bioinformatics Online.

[79] Excoffier L, Smouse P, Quattro J (1992). Analysis of molecular variance inferred from metric distances among DNA haplotypes: application to human mitochondrial DNA restriction data. Genetics.

[80] Heled J, Drummond AJ (2010). Bayesian inference of species trees from multilocus data. Mol Biol Evol.

[81] Fu YX (1997). Statistical neutrality of mutations against population growth, hitchhiking and background selection. Genetics.

[82] Tajima F (1989). Statistical-method for testing the neutral mutation hypothesis by DNA polymorphism. Genetics.

[83] Harpending RC (1994). Signature of ancient population growth in a low-resolution mitochondrial DNA mismatch distribution. Hum Biol.

[84] Schneider S, Excoffier L (1999). Estimation of demographic parameters from the distribution of pairwise differences when the mutation rates vary among sites: application to human mitochondrial DNA. Genetics.

[85] Robert CP, Cornuet JM, Marin JM, Pillai NS (2011). Lack of confidence in approximate Bayesian computation model choice. Proc Natl Acad Sci U S A.

[86] Rogers AR, Harpending H (1992). Population growth makes waves in the distribution of pairwise differences. Mol Biol Evol.

[87] Drummond AJ, Rambaut A, Shapiro B, Pybus OG (2005). Bayesian coalescent inference of past population dynamics from molecular sequences. Mol Biol Evol.

[88] Hey J, Nielsen R (2007). Integration within the Felsenstein equation for improved Markov chain Monte Carlo methods in population genetics. Proc Natl Acad Sci U S A.

[89] Hey J, Nielsen R (2004). Multilocus methods for estimating population sizes, migration rates and divergence time, with applications to the divergence of *Drosophila pseudoobscura* and *D. persimilis*. Genetics.

[90] Lande R, Engen S, Sæther BE (2003). Stochastic population dynamics in ecology and conservation.

[91] Cornuet JM, Santos F, Beaumont MA, Robert CP, Marin JM, Balding DJ, Guillemaud T, Estoup A (2008). Inferring population history with DIY ABC: a user-friendly approach to approximate Bayesian computation. Bioinformatics.

[92] Cornuet JM, Pudlo P, Veyssier J, Dehne-Garcia A, Gautier M, Leblois R, Marin JM, Estoup A (2014). DIYABC v2.0: a software to make approximate Bayesian computation inferences about population history using single nucleotide polymorphism, DNA sequence and microsatellite data. Bioinformatics.

[93] Fontaine MC, Austerlitz F, Giraud T, Labbé F, Papura D, Richard-Cervera S, Delmotte F (2013). Genetic signature of a range expansion and leap-frog event after the recent invasion of Europe by the grapevine downy mildew pathogen *Plasmopara viticola*. Mol Ecol.

[94] Barrier E, Velasquillo L, Chávez M, Gaulon R (1998). Neotectonic evolution of the Isthmus of Tehuantepec (southeastern Mexico). Tectonophysics.

[95] Manea M, Manea VC, Ferrari L, Kostoglodov V, Bandy WL (2005). Tectonic evolution of the Tehuantepec ridge. Earth Planet Sci Lett.

[96] Barber BR, Klicka J (2010). Two pulses of diversification across the Isthmus of Tehuantepec in a montane Mexican bird fauna. Proc R Soc Lond B Biol Sci.

[97] Daza JM, Castoe TA, Parkinson CL (2010). Using regional comparative phylogeographic data from snake lineages to infer historical processes. Ecography.

[98] Gutiérrez-Rodríguez C, Ornelas JF, Rodríguez-Gómez F (2011). Chloroplast DNA phylogeography of a distylous shrub (*Palicourea padifolia*, Rubiaceae) reveals allopatric fragmentation and demographic expansion in Mexican cloud forests. Mol Phylogenet Evol.

[99] Jaramillo-Correa JP, Beaulieu J, Khasa DP, Bousquet J (2009). Inferring the past from the present phylogeographic structure of North American forest trees: seeing the forest for the genes. Can J Plant Res.

[100] Ornelas JF, Ruiz-Sanchez E, Sosa V (2010). Phylogeography of *Podocarpus matudae* (Podocarpaceae): pre-Quaternary age relicts in the northern Mesoamerican cloud forests. J Biogeogr.

[101] Twyford AD, Kidner CA, Harrison N, Ennos RA (2013). Population history and seed dispersal in widespread Central American *Begonia* species (Begoniaceae) inferred from plastome-derived microsatellite markers. Bot J Linn Soc.

[102] Smith CI, Drummond CS, Godsoe W, Yoder JB, Pellmyr O (2009). Host specificity and reproductive success of yucca moths (*Tegeticula* spp. Lepidoptera: Prodoxidae) mirror patterns of gene flow between host plant varieties of the Joshua tree (*Yucca brevifolia*: Agavaceae). Mol Ecol.

[103] González C, Harvey N, Ornelas JF (2015). Development and characterization of microsatellite loci in the mistletoe *Psittacanthus schiedeanus* (Loranthaceae). Appl Plant Sci.

[104] Ornelas JF, Gándara E, Vásquez-Aguilar AA, Ramírez-Barahona S, Ortiz-Rodríguez AE, González C, Mejía Saules MT, Ruiz-Sanchez E. Data from: a mistletoe tale: Postglacial invasion of *Psittacanthus schiedeanus* (Loranthaceae) to Mesoamerican cloud forests revealed by molecular data and species distribution modeling. 2016. Dryad Digital Repository, http://dx.doi.org/10.5061/dryad.t49h6. 10.1186/s12862-016-0648-6 PMC4830056 27071983

