# Supplementary figures and images for: A mistletoe tale: postglacial invasion of Psittacanthus schiedeanus (Loranthaceae) to Mesoamerican cloud forests revealed by molecular data and species distribution modeling
Source: BMC Evol Biol. 2016 Apr 12;16:78. doi: 10.1186/s12862-016-0648-6 (PMC4830056; doi:10.1186/s12862-016-0648-6)

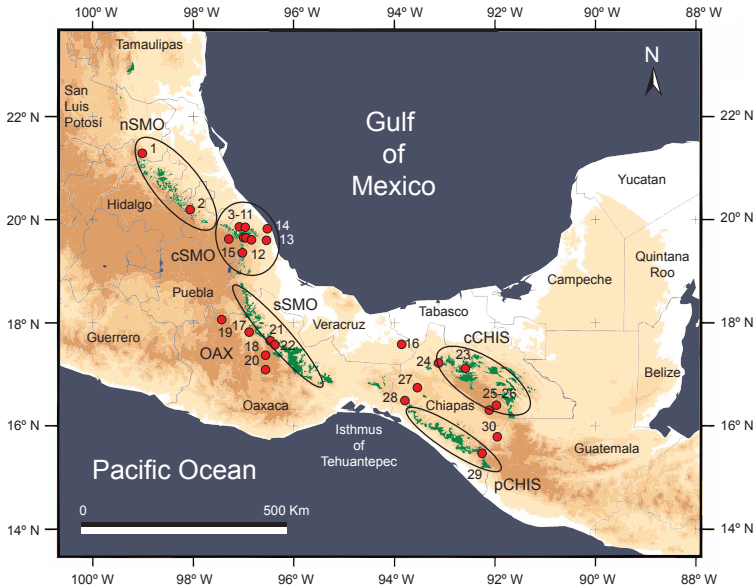

Supplement: Additional file 1: — Map showing collecting sites of Psittacanthus schiedeanus. Numbers refer to collection sites and thin ellipses show collecting sites within the cloud forest areas. Region abbreviations are as follows: nSMO northern Sierra Madre Oriental, cSMO central Sierra Madre Oriental, sSMO southern Sierra Madre Oriental, CHIS Chiapan Highlands (cCHIS and pCHIS) separated by the Central Depression that together with Guatemala form the region Trans-Isthmian Highlands (TIH), OAX Oaxacan drylands. (PDF 5098 kb) [file 12862_2016_648_MOESM1_ESM.pdf]

(a)

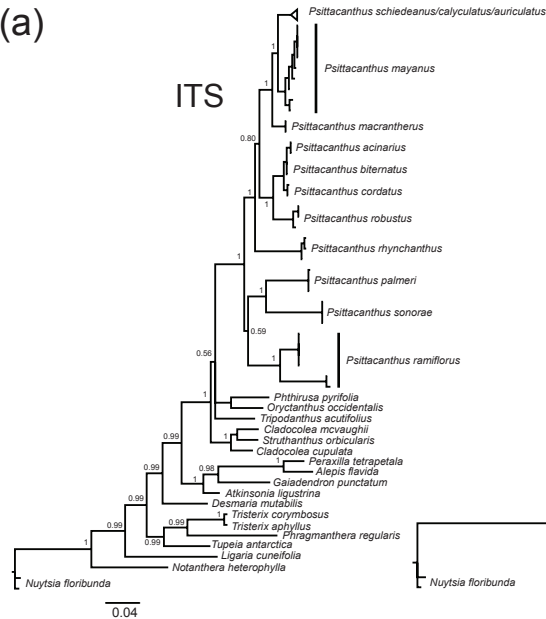

(b)

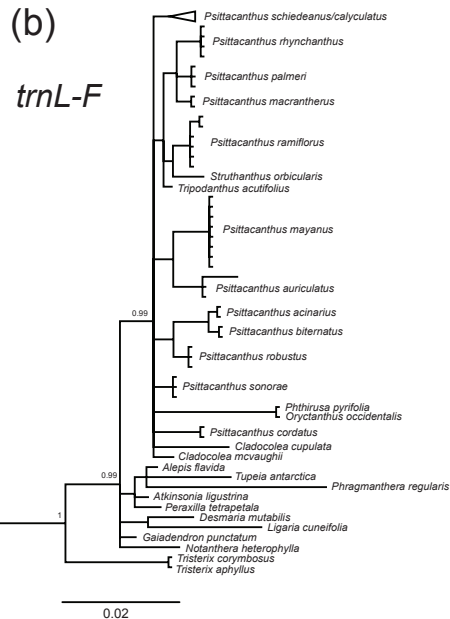

(c)

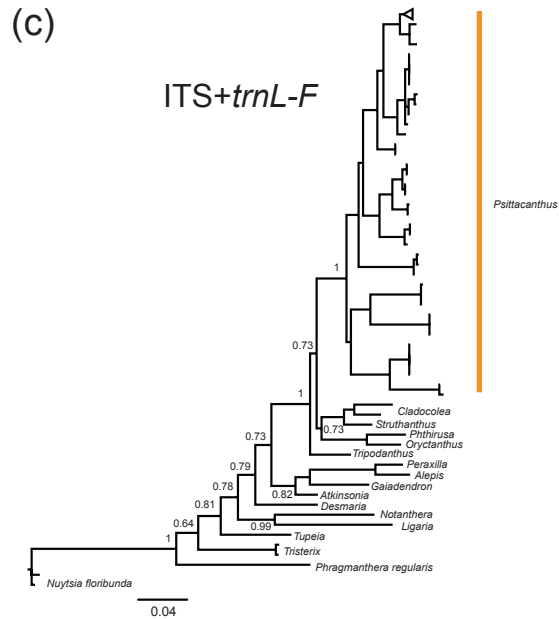

(d)

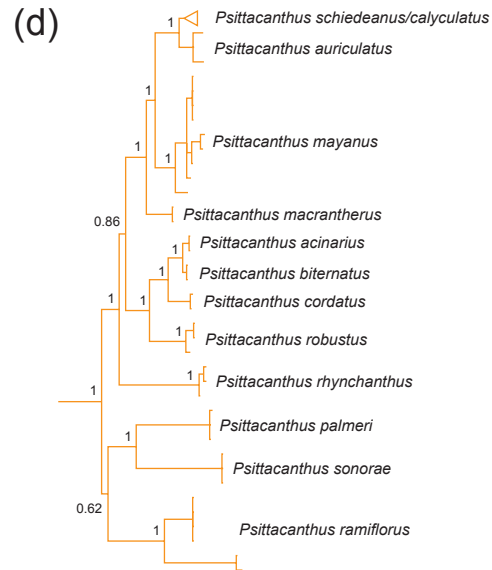

Supplement: Additional file 4: — Phylogenetic trees of ITS, trnL-F and ITS+trnL-F. Bayesian posterior probabilities for MrBayes analyses. Illustrations of tree topologies based on (a) ITS, (b) trnL-F, and (c) concatenated sequences for samples of the Psittacanthus clade. The values above branches denote posterior probabilities (PP). The distal portion of the phylogeny based on concatenated sequences is shown, representing the Psittacanthus schiedeanus samples through other Psittacanthus species (d). (PDF 325 kb) [file 12862_2016_648_MOESM4_ESM.pdf]
