# Supplementary material for: A mistletoe tale: postglacial invasion of Psittacanthus schiedeanus (Loranthaceae) to Mesoamerican cloud forests revealed by molecular data and species distribution modeling
Source: BMC Evol Biol. 2016 Apr 12;16:78. doi: 10.1186/s12862-016-0648-6 (PMC4830056; doi:10.1186/s12862-016-0648-6)
Supplement: Additional file 2: — Taxon sampling. Voucher information, geographic location and population code of the 31 Psittacanthus schiedeanus populations used in the study. IDs reported refer to accession numbers in the Instituto de Ecología, AC (XAL) herbarium. (DOC 150 kb) [file 12862_2016_648_MOESM2_ESM.doc]

**Additional file 2 Voucher information, geographic location and population code of the 31 *Psittacanthus schiedeanus* populations used in the study. IDs reported refer to accession numbers in the Instituto de Ecología, AC (XAL) herbarium.**

| Location Code | Location | Region* | *n* | Altitude  (m asl) | Latitude  (N) | Longitude (W) | Voucher information |
| --- | --- | --- | --- | --- | --- | --- | --- |
|  |  |  |  |  |  |  |  |
| 1 | Mexico, San Luis Potosí, Xilitla | nSMO | 10 | 836 | 21° 21´ 39´´ | 98° 59´ 35´´ | E. Ruiz 281 (XAL) |
| 2 | Mexico, Puebla, Lagunillas | nSMO | 8 | 1500 | 20° 13´ 23´´ | 97° 57´ 33´´ | E. Ruiz s/n (XAL) |
| 3 | Mexico, Veracruz, Clavijero | cSMO | 16 | 1225 | 19° 30´ 47´´ | 96° 56´ 28´´ | M.T. Mejía 2036 (XAL) |
| 4 | Mexico, Veracruz, La Pitaya | cSMO | 15 | 1343 | 19º 30´ 27´´ | 96º 57´ 39´´ | M.T. Mejía 2035 (XAL) |
| 5 | Mexico, Veracruz, El Riscal | cSMO | 13 | 1586 | 19º 28´ 47´´ | 96º 59´ 51´´ | M.T. Mejía 2044 (XAL) |
| 6 | Mexico, Veracruz, Coapexpan | cSMO | 12 | 1392 | 19º 31´ 22´´ | 96º 58´ 02´´ | M.T. Mejía 2038 (XAL) |
| 7 | Mexico, Veracruz, Rancho Viejo | cSMO | 13 | 1350 | 19º 31´ 11´´ | 96º 58´ 22´´ | M.T. Mejía 2039 (XAL) |
| 8 | Mexico, Veracruz, Xoloxtla | cSMO | 12 | 1454 | 19º 31´ 36´´ | 97º 00´ 34´´ | M.T. Mejía 2040 (XAL) |
| 9 | Mexico, Veracruz, Tlalnelhuayocan | cSMO | 10 | 1624 | 19º 34´ 47´´ | 96º 57´ 38´´ | M.T. Mejía 2041 (XAL) |
| 10 | Mexico, Veracruz, Coacoatzintla | cSMO | 12 | 1501 | 19º 37´ 41´´ | 96º 52´ 56´´ | M.T. Mejía 2043 (XAL) |
| 11 | Mexico, Veracruz, Xico | cSMO | 17 | 1350 | 19º 24´ 37´´ | 96º 59´ 31´´ | M.T. Mejía 2037 (XAL) |
| 12 | Mexico, Veracruz, Volcán de Acatlán | cSMO | 11 | 1840 | 19º 40´ 47´´ | 96º 51´ 11´´ | M.T. Mejía 2042 (XAL) |
| 13 | Mexico, Veracruz, Actópan | cSMO | 10 | 322 | 19º 23´ 13´´ | 96º 36´ 56´´ | M.T. Mejía 2049 (XAL) |
| 14 | Mexico, Veracruz, Cardel, La Mancha | cSMO | 7 | 61 | 19° 36´ 02´´ | 96° 22´ 29´´ | M.T. Mejía 2050 (XAL) |
| 15 | Mexico, Veracruz, Las Minas | cSMO | 10 | 1828 | 19º 34´ 17´´ | 96º 59´ 21´´ | M.T. Mejía 2057 (XAL) |
| 16 | Mexico, Veracruz, Las Choapas | cSMO | 2 | 18 | 17° 38´ 41´´ | 93° 58´ 54´´ | M.T. Mejía 2059 (XAL) |
| 17 | Mexico, Oaxaca, Puente Xia | CALY | 8 | 1513 | 17° 18´ 19´´ | 96° 31´ 35´´ | E. Ruiz 439 (XAL) |
| 18 | Mexico, Oaxaca, Tuxtepec, Ixtlán | CALY | 10 | 1943 | 17° 14´ 28´´ | 96° 29´ 09´´ | E. Ruiz 438 (XAL) |
| 19 | Mexico, Oaxaca, Huajuapan, La Presa | CALY | 10 | 1869 | 17° 41´ 41´´ | 97° 36´ 01´´ | - |
| 20 | Mexico, Oaxaca, Col. Emiliano Zapata | CALY | 7 | 1484 | 16° 45´ 07´´ | 96° 50´ 42´´ | E. Ruiz 334 (XAL) |
| 21 | Mexico, Oaxaca, Comaltepec, Metates | sSMO | 10 | 848 | 17° 41´ 23´´ | 96° 20´ 13´´ | A. Ortíz-Rodríguez 754 (XAL) |
| 22 | Mexico, Oaxaca, Comaltepec, Pto Eligio | sSMO | 2 | 710 | 17° 42´ 14´´ | 96° 18´ 26´´ | A. Ortíz-Rodríguez 755 (XAL) |
| 23 | Mexico, Chiapas, Ocosingo | CHIS | 3 | 876 | 16° 55´ 06´´ | 92° 06´ 09´´ | E. Gándara 3109 (XAL) |
| 24 | Mexico, Chiapas, Jitotol | CHIS | 6 | 1698 | 17° 02´ 54´´ | 92° 51´ 18´´ | E. Ruiz 263 (XAL) |
| 25 | Mexico, Chiapas, Comitán | CHIS | 5 | 1630 | 16° 13´ 20´´ | 92° 07´ 52´´ | A. Ortíz-Rodríguez 807 (XAL) |
| 26 | Mexico, Chiapas, Parque Ya’ Ax-Na | CHIS | 5 | 1595 | 16° 13´ 24´´ | 92° 07´ 48´´ | E. Gándara 3099 (XAL) |
| 27 | Mexico, Chiapas, Ocozocuautla, Arriaga | CHIS | 5 | 667 | 16° 24´ 24´´ | 93° 48´ 22´´ | E. Ruiz 259 (XAL) |
| 28 | Mexico, Chiapas, Arriaga, La Aurora | BREE | 5 | 699 | 16° 28´ 18´´ | 93° 49´ 55´´ | E. Gándara 3089-91 (XAL) |
| 29 | Mexico, Chiapas, Motozintla | CHIS | 7 | 1353 | 15° 21´ 21´´ | 92° 14´ 54´´ | E. Ruiz 261 (XAL) |
| 30 | Mexico, Chiapas, Ciudad Cuauhtemoc | BREE | 10 | 1834 | 15° 55´ 04´´ | 91° 58´ 55´´ | E. Ruiz 262 (XAL) |
| 31 | Panama, Chiriquí | CHIS | 3 | - | - | - | - |
|  |  |  |  |  |  |  |  |

* nSMO = northern Sierra Madre Oriental; cSMO = central Sierra Madre Oriental; sSMO = southern Sierra Madre Oriental; CALY = central Oaxaca, CHIS = Chiapan highlands separated by the Central Depression, and BREE = Central Depression, Chiapas.

.
