# Supplementary material for: A mistletoe tale: postglacial invasion of Psittacanthus schiedeanus (Loranthaceae) to Mesoamerican cloud forests revealed by molecular data and species distribution modeling
Source: BMC Evol Biol. 2016 Apr 12;16:78. doi: 10.1186/s12862-016-0648-6 (PMC4830056; doi:10.1186/s12862-016-0648-6)
Supplement: Additional file 3: — Outgroup sampling. Geographic location, GenBank accession numbers and voucher information of Psittacanthus species used in this study. IDs reported refer to accession numbers in the Instituto de Ecología, AC (XAL) and Universidade de São Pablo (USP) herbaria. (DOC 83 kb) [file 12862_2016_648_MOESM3_ESM.doc]

**Additional file 3 Geographic location, GenBank accession numbers and voucher information of *Psittacanthus* species used in this study. IDs reported refer to de accession numbers in the Instituto Ecología, AC (XAL) and Universidade de São Pablo (USP) herbaria. MX: Mexico, BR = Brazil, GT = Guatemala.**

| Species | Location | Latitude | Longitude | ITS | *trn*L*-*F | Voucher information |
| --- | --- | --- | --- | --- | --- | --- |
|  |  |  |  |  |  |  |
| *P. acinarius* | BR: Mato Grosso, Cuiabá | –15º 35´ 56´´ | –56º 05´ 42´´ | KU923005 | KU923279 | G. Ceccantini 3670A (USP) |
| *P. acinarius* | BR: Mato Grosso, Cuiabá | –15º 35´ 56´´ | –56º 05´ 42´´ | KU923006 | KU923280 | G. Ceccantini 3670B (USP) |
| *P. auriculatus* | MX: Oaxaca, El Molino | 17º 46´ 14´´ | –97º 44´ 58´´ | KU923007 | KU923281 | M. J. Pérez 004 (XAL) |
| *P. auriculatus* | MX: Oaxaca, El Molino | 17º 46´ 14´´ | –97º 44´ 58´´ | KU923008 | KU923282 | M. J. Pérez 005 (XAL) 016 000000000000000000000000000000000000000000000000000000000000000000000000000000000000000000000000000000000000000000000000000 |
| *P. auriculatus* | MX: Oaxaca, El Molino | 17º 46´ 14´´ | –97º 44´ 58´´ | KU923009 | KU923283 | - |
| *P. biternatus* | BR: Mato Grosso | –15º 35´ 56´´ | –56º 05´ 42´´ | KU923010 | KU923284 | G. Ceccantini 3785 (USP) |
| *P. biternatus* | BR: Mato Grosso | –15º 35´ 56´´ | –56º 05´ 42´´ | KU923011 | KU923285 | G. Ceccantini 3786 (USP) |
| *P. calyculatus* | MX: Michoacán, Maravatío | 19º 54´ 00´´ | –93º 49´ 47´´ | KU923012 | KU923286 | E. Ruiz-Sanchez 414 (XAL) |
| *P. calyculatus* | MX: Michoacán, Maravatío | 19º 54´ 00´´ | –100º 27´ 00´´ | KU923013 | KU923287 | E. Ruiz-Sanchez 415 (XAL) |
| *P. calyculatus* | MX: Michoacán, Morelia | 19º 60´ 05´´ | –101º 23´ 00´´ | KU923014 | KU923288 | - |
| *P. calyculatus* | MX: Tlaxcala, Tlaxcala | 19º 17´ 00´´ | –98º 14´ 00´´ | KU923015 | KU923289 | - |
| *P. calyculatus* | MX: Tlaxcala, Tlaxcala | 19º 17´ 00´´ | –98º 14´ 00´´ | KU923016 | KU923290 | - |
| *P. calyculatus* | MX: Jalisco, San José de Gracia | 20° 41´ 47´´ | 102° 33´ 22´´ | KU923017 | KU923291 | E. Ruiz-Sanchez 307 (XAL) |
| *P. calyculatus* | MX: Jalisco, San José de Gracia | 20° 41´ 47´´ | 102° 33´ 22´´ | KU923018 | KU923292 | - |
| *P. cordatus* | BR: Mato Grosso, Cuiabá | –15º 35´ 56´´ | –56º 05´ 42´´ | KU923019 | KU923293 | G. Ceccantini 3671 (USP) |
| *P. cordatus* | BR: Mato Grosso, Cuiabá | –15º 35´ 56´´ | –56º 05´ 42´´ | KU923020 | KU923294 | G. Ceccantini 3672 (USP) |
| *P. macrantherus* | MX: Sinaloa, El Palmito | 23º 33´ 00´´ | –105º 50´ 00´´ | KU923021 | KU923295 | E. Ruiz-Sanchez 348 (XAL) |
| *P. macrantherus* | MX: Sinaloa, El Palmito | 23º 33´ 00´´ | –105º 50´ 00´´ | KU923022 | KU923296 | F. Rodríguez s/n (XAL) |
| *P. mayanus* | MX: Yucatán, Hunucmá | 21º 02´ 58´´ | –89º 54´ 38´´ | KU923023 | KU923297 | - |
| *P. mayanus* | MX: Yucatán, Hunucmá | 21º 02´ 58´´ | –89º 54´ 38´´ | KU923024 | KU923298 | - |
| *P. mayanus* | MX: Yucatán, Cuxtal | 20º 54´ 37´´ | –89º 37´ 15´´ | KU923025 | KU923299 | - |
| *P. mayanus* | MX: Yucatán, Cuxtal | 20º 54´ 37´´ | –89º 37´ 15´´ | KU923026 | KU923300 | - |
| *P. mayanus* | MX: Chiapas, Ocozocuautla | 16º 47´ 47´´ | –93º 24´ 30´´ | KU923027 | KU923301 | Y. Licona Vera 017 (XAL) |
| *P. mayanus* | MX: Chiapas, Ocozocuautla | 16º 47´ 47´´ | –93º 24´ 30´´ | KU923028 | KU923302 | Y. Licona Vera 018 (XAL) |
| *P. palmeri* | MX: Oaxaca, Cañón del Sabino | 17º 51´ 53´´ | –97º 02´ 10´´ | KU923029 | KU923303 | - |
| *P. palmeri* | MX: Oaxaca, Cañón del Sabino | 17º 51´ 53´´ | –97º 02´ 10´´ | KU923030 | KU923304 | - |
| *P. palmeri* | MX: Oaxaca, Cañón del Sabino | 17º 51´ 53´´ | –93º 24´ 30´´ | KU923031 | KU923305 | - |
| *P. ramiflorus* | MX: Chiapas, Berriozabal | 16º 50´ 21´´ | –93º 18´ 11´´ | KU923032 | KU923306 | Y. Licona Vera 013 (XAL) |
| *P. ramiflorus* | MX: Chiapas, Berriozabal | 16º 50´ 21´´ | –93º 18´ 11´´ | KU923033 | KU923307 | Y. Licona Vera 014 (XAL) |
| *P. ramiflorus* | MX: Chiapas, Berriozabal | 16º 50´ 21´´ | –93º 18´ 11´´ | KU923034 | KU923308 | Y. Licona Vera 015 (XAL) |
| *P. ramiflorus* | MX: Chiapas, Berriozabal | 16º 50´ 21´´ | –93º 18´ 11´´ | KU923035 | KU923309 | Y. Licona Vera 016 (XAL) |
| *P. rhynchanthus* | GT: Patulul, San Salvador | 14º 22´ 24´´ | –91º 08´ 18´´ | KU923038 | KU923312 | P. Carrillo Reyes 5372 (XAL) |
| *P. rhynchanthus* | GT: Patulul, Santa Fé | 14º 27´ 07´´ | –91º 08´ 30´´ | KU923039 | KU923313 | P. Carrillo Reyes 5374 (XAL) |
| *P. rhynchanthus* | MX: Michoacán, La Peña | 18º 10´ 43´´ | –102º 28´ 08´´ | KU923040 | KU923314 | E. Ruiz-Sanchez 417 (XAL) |
| *P. rhynchanthus* | MX: Veracruz, Pinoltepec | 19º 26´ 01´´ | –96º 42´ 15´´ | KU923041 | KU923315 | T. Mejía-Saules 2048 (XAL) |
| *P. robustus* | BR: Minas Gerais, Serra do Cipó | –19º 18´ 26´´ | –43º 52´ 33´´ | KU923042 | KU923316 | G. Ceccantini 3588 (USP) |
| *P. robustus* | BR: Minas Gerais, Serra do Cipó | –19º 18´ 26´´ | –43º 52´ 33´´ | KU923043 | KU923317 | G. Ceccantini 3589 (USP) |
| *P. robustus* | BR: Minas Gerais, Serra do Cipó | –19º 18´ 26´´ | –43º 52´ 33´´ | KU923044 | KU923318 | G. Ceccantini 3596 (USP) |
| *P. sonorae* | MX: Sonora, Nacapule | 27º 59´ 04´´ | –111º 02´ 40´´ | KU923045 | KU923319 | - |
| *P. sonorae* | MX: Sonora, Cruz de Piedra | 27º 57´ 25´´ | –110º 40´ 51´´ | KU923046 | KU923320 | - |
| *P. sonorae* | MX: Sonora, Paraíso La Manga | 27º 53´ 43´´ | –111º 06´ 55´´ | KU923047 | KU923321 | - |
|  |  |  |  |  |  |  |
