# Supplementary material for: A mistletoe tale: postglacial invasion of Psittacanthus schiedeanus (Loranthaceae) to Mesoamerican cloud forests revealed by molecular data and species distribution modeling
Source: BMC Evol Biol. 2016 Apr 12;16:78. doi: 10.1186/s12862-016-0648-6 (PMC4830056; doi:10.1186/s12862-016-0648-6)
Supplement: Additional file 5: — Haplotype information. Number of genetically analyzed samples (n) for each molecular marker (ITS and trnL-F) and number of distinct ribotypes (R) and haplotypes (H) found in Psittacanthus schiedeanus individuals sampled, and the number of individuals per ribotype or haplotype in parentheses. Codes are from networks in Figs. 4 and 5. (DOC 73 kb) [file 12862_2016_648_MOESM5_ESM.doc]

**Additional file 5 Number of genetically analyzed samples (*n*) for each molecular marker (ITS and *trnL-F*) and number of distinct ribotypes (R) and haplotypes (H) found in *Psittacanthus schiedeanus* individuals sampled, and the number of individuals per ribotype or haplotype in parentheses. Codes are from networks in Figs 4 and 5.**

| Location Code | Location | Habitat type* | *n* | ITS ribotype | *n* | *trnL-F* haplotype |
| --- | --- | --- | --- | --- | --- | --- |
|  |  |  |  |  |  |  |
| 1 | Mexico, San Luis Potosí, Xilitla | SCHI | 7 | R1(3), R2(1), R5(2), R40(1) | 8 | H3(8) |
| 2 | Mexico, Puebla, Lagunillas | SCHI | 7 | R1(1), R2(4), R5(2) | 5 | H3(5) |
| 3 | Mexico, Veracruz, Clavijero | SCHI | 15 | R2(1), R5(5), R12(1), R13(2), R14(1), R28(2), R40(3) | 15 | H3(15) |
| 4 | Mexico, Veracruz, La Pitaya | SCHI | 15 | R5(4), R7(1), R8(1), R9(1), R10(1), R11(1), R19(1), R23(3), R25(1), R29(1) | 13 | H1(1), H3(12) |
| 5 | Mexico, Veracruz, El Riscal | SCHI | 13 | R3(1), R5(10), R6(1), R23(1) | 13 | H2(2), H3(11) |
| 6 | Mexico, Veracruz, Coapexpan | SCHI | 11 | R4(1), R5(2), R23(3), R24(2), R27(1), R28(1), R44(1) | 12 | H3(12) |
| 7 | Mexico, Veracruz, Rancho Viejo | SCHI | 13 | R5(6), R30(4), R39(2), R43(1) | 12 | H2(1), H3(9), H4(1), H5(1) |
| 8 | Mexico, Veracruz, Xoloxtla | SCHI | 12 | R5(10), R12(2) | 11 | H3(11) |
| 9 | Mexico, Veracruz, Tlalnelhuayocan | SCHI | 7 | R5(4), R16(1), R32(2) | 9 | H3(9) |
| 10 | Mexico, Veracruz, Coacoatzintla | SCHI | 11 | R1(1), R2(1), R5(4), R23(5) | 11 | H2(1), H3(10) |
| 11 | Mexico, Veracruz, Xico | SCHI | 14 | R5(10), R35(2), R41(1), R42(1) | 15 | H3(15) |
| 12 | Mexico, Veracruz, Volcán de Acatlán | SCHI | 10 | R5(7), R12(1), R23(1), R38(1) | 8 | H3(8) |
| 13 | Mexico, Veracruz, Actópan | SCHI | 10 | R5(9), R16(1) | 10 | H3(10) |
| 14 | Mexico, Veracruz, Cardel, La Mancha | SCHI | 7 | R5(5), R17(1), R18(1) | 6 | H3(6) |
| 15 | Mexico, Veracruz, Las Minas | SCHI | 10 | R5(8), R32(2) | 6 | H3(2), H6(4) |
| 16 | Mexico, Veracruz, Las Choapas | SCHI | - | - | 2 | H3(1), H9(1) |
| 17 | Mexico, Oaxaca, Tuxtepec, Puente Xia | CALY | 8 | R5(8) | 8 | H7(8) |
| 18 | Mexico, Oaxaca, Tuxtepec, Ixtlán | CALY | 8 | R5(3), R19(3), R21(2) | 6 | H7(6) |
| 19 | Mexico, Oaxaca, Huajuapan, La Presa | CALY | 10 | R5(3), R23(4), R26(3) | 10 | H8(10) |
| 20 | Mexico, Oaxaca, Col. Emiliano Zapata | CALY | 7 | R5(3), R26(2), R33(2) | 7 | H7(2), H9(5) |
| 21 | Mexico, Oaxaca, Comaltepec, Metates | SCHI | 10 | R23(10) | 10 | H3(10) |
| 22 | Mexico, Oaxaca, Comaltepec, Pto. Eligio | SCHI | 2 | R23(2) | 2 | H3(2) |
| 23 | Mexico, Chiapas, Ocosingo | SCHI | 3 | R35(1), R36(2) | 3 | H3(2), H7(1) |
| 24 | Mexico, Chiapas, Jitotol | SCHI | 7 | R35(5), R36(2) | 6 | H3(6) |
| 25 | Mexico, Chiapas, Comitán | SCHI | 5 | R5(3), R19(1), R20(1) | 5 | H3(3), H9(2) |
| 26 | Mexico, Chiapas, Parque Ya’ Ax-Na | SCHI | 5 | R19(4), R22(1) | 5 | H3(5) |
| 27 | Mexico, Chiapas, Ocozocuautla, Arriaga | SCHI | - | - | 1 | H3(1) |
| 28 | Mexico, Chiapas, Arriaga, La Aurora | BREE | 5 | R5(2), R15(1), R23(2) | 5 | H9(5) |
| 29 | Mexico, Chiapas, Motozintla | SCHI | 8 | R5(2), R34(1), R35(4), R37(1) | 6 | H3(6) |
| 30 | Mexico, Chiapas, Ciudad Cuauhtemoc | BREE | 10 | R30(1), R31(9) | 10 | H9(10) |
| 31 | Panama, Chiriquí | SCHI | 1 | R5(1) | 1 | H3(1) |
|  |  |  |  |  |  |  |

* Habitat type abbreviations are as follows: SCHI = cloud forests from San Luis Potosí to Oaxaca and Chiapas, CALY = xeric vegetation in central Oaxaca, BREE = tropical deciduous forests in Chiapas.
