# Supplementary material for: A mistletoe tale: postglacial invasion of Psittacanthus schiedeanus (Loranthaceae) to Mesoamerican cloud forests revealed by molecular data and species distribution modeling
Source: BMC Evol Biol. 2016 Apr 12;16:78. doi: 10.1186/s12862-016-0648-6 (PMC4830056; doi:10.1186/s12862-016-0648-6)
Supplement: Additional file 6: — Summary statistics. Summary statistics of demographic analysis of Psittacanthus schiedeanus samples by habitat type to infer demographic range expansion. (DOC 39 kb) [file 12862_2016_648_MOESM6_ESM.doc]

**Additional file 6 Summary statistics of demographic analysis of *Psittacanthus schiedeanus* samples by habitat type to infer demographic range expansion.**

|  |  |  |  | |  | ITS | |  | |  | |  | |  | |  | |  | |  | |  | | *trnL-F* | |  | |  | |  | |  | |
| --- | --- | --- | --- | --- | --- | --- | --- | --- | --- | --- | --- | --- | --- | --- | --- | --- | --- | --- | --- | --- | --- | --- | --- | --- | --- | --- | --- | --- | --- | --- | --- | --- | --- |
| Group | *N* | *N_H_* | *h* | | π | | *D*_T_ | | *F_S_* | | *SSD* | | *Hri* | |  | | *N* | | *N_H_* | | *h* | | π | | *D*_T_ | | *F_S_* | | *SSD* | | *Hri* | |  |
|  |  | | |  | | | | | | | | | | | | | | | | | | | | | | | | | | | | | |
| SCHI | 203 | 24 | 0.41±0.04 | | 0.0016±0.001 | | **–2.312**** | | **–25.865***** | | 0.2369*** | | **0.3039** | |  | | 192 | | 7 | | 0.14±0.03 | | 0.0088±0.005 | | **–2.083***** | | 3.282 | | **0.0060** | | **0.6647** | |  |
| BREE | 14 | 1 | 0.00±0.00 | | 0.0000±0.000 | | n.a. | | n.a. | | n.a. | | n.a. | |  | | 15 | | 1 | | 0.00±0.00 | | 0.0000±0.000 | | n.a. | | n.a. | | n.a. | | n.a. | |  |
| CALY | 33 | 3 | 0.23±0.09 | | 0.0008±0.001 | | –1.311 | | –0.0001 | | 0.0614* | | **0.6938** | |  | | 31 | | 3 | | 0.62±0.05 | | 0.0198±0.011 | | 1.134 | | 10.617 | | 0.2157* | | 0.4607*** | |  |
|  |  |  |  | |  | |  | |  | |  | |  | |  | |  | |  | |  | |  | |  | |  | |  | |  | | |

*N* = number of individuals, *N_H_* = number of ribotypes or haplotypes, *h* = gene diversity, π = nucleotide diversity, *D*_T_ = Tajima’s *D*, *F_S_* = Fu’s *F*s, *SDD* = differences in the sum of squares or mismatch distribution, *Hri* = Harpending’s raggedness index. n.a. = not available; **P* < 0.05; ***P* < 0.01; ****P* < 0.001; *****P* < 0.0001. *D*_T_ and *F_S_* positive values are indicative of mutation-drift-equilibrium, which is typical of stable populations, and negative values that result from an excess of rare haplotypes indicate that populations have undergone recent expansions, often preceded by a bottleneck. Significantly negative values (at the 0.05 level) reveal in both tests historic demographic expansion events. Significant (*P* ≤ 0.05) *SSD* and *Hri* values indicate deviations from the sudden expansion model. In bold are shown values that are consistent with demographic expansion. Habitat type abbreviations are as follows: SCHI = cloud forests from San Luis Potosí to Oaxaca and Chiapas, CALY = xeric vegetation in central Oaxaca, BREE = tropical deciduous forests in Chiapas.
